# Oligodendrocytes produce amyloid-β and contribute to plaque formation alongside neurons in Alzheimer’s disease model mice

**DOI:** 10.1038/s41593-024-01730-3

**Published:** 2024-08-05

**Authors:** Andrew Octavian Sasmita, Constanze Depp, Taisiia Nazarenko, Ting Sun, Sophie B. Siems, Erinne Cherisse Ong, Yakum B. Nkeh, Carolin Böhler, Xuan Yu, Bastian Bues, Lisa Evangelista, Shuying Mao, Barbara Morgado, Zoe Wu, Torben Ruhwedel, Swati Subramanian, Friederike Börensen, Katharina Overhoff, Lena Spieth, Stefan A. Berghoff, Katherine Rose Sadleir, Robert Vassar, Simone Eggert, Sandra Goebbels, Takashi Saito, Takaomi Saido, Gesine Saher, Wiebke Möbius, Gonçalo Castelo-Branco, Hans-Wolfgang Klafki, Oliver Wirths, Jens Wiltfang, Sarah Jäkel, Riqiang Yan, Klaus-Armin Nave

**Affiliations:** 1 Department of Neurogenetics, Max Planck Institute for Multidisciplinary Sciences, Göttingen, Germany; 2 International Max Planck Research School for Neurosciences, Göttingen, Germany; 3 Laboratory of Molecular Neurobiology, Department of Biochemistry and Biophysics, Karolinska Institutet, Stockholm, Sweden; 4 School of Biochemistry and Cell Biology, Biosciences Institute, University College Cork, Cork, Ireland; 5 Institute for Stroke and Dementia Research, Klinikum Der Universität München, Ludwig-Maximilians-Universität, Munich, Germany; 6 Department of Psychiatry and Psychotherapy, University Medical Center, Georg-August University, Göttingen, Germany; 7 Electron Microscopy Core Unit, Max Planck Institute Multidisciplinary Sciences, Göttingen, Germany; 8 Ken and Ruth Davee Department of Neurology, Northwestern University Feinberg School of Medicine, Chicago, IL USA; 9 Mesulam Center for Cognitive Neurology and Alzheimer’s Disease, Northwestern University Feinberg School of Medicine, Chicago, IL USA; 10 Laboratory for Proteolytic Neuroscience, RIKEN Center for Brain Science Wako, Saitama, Japan; 11 German Center for Neurodegenerative Diseases (DZNE), Göttingen, Germany; 12 Munich Cluster for System Neurology (SyNergy), Munich, Germany; 13 Department of Neuroscience, UConn Health, Farmington, CT USA

**Keywords:** Alzheimer's disease, Oligodendrocyte

## Abstract

Amyloid-β (Aβ) is thought to be neuronally derived in Alzheimer’s disease (AD). However, transcripts of amyloid precursor protein (*APP*) and amyloidogenic enzymes are equally abundant in oligodendrocytes (OLs). By cell-type-specific deletion of *Bace1* in a humanized knock-in AD model, *APP*^*NLGF*^, we demonstrate that OLs and neurons contribute to Aβ plaque burden. For rapid plaque seeding, excitatory projection neurons must provide a threshold level of Aβ. Ultimately, our findings are relevant for AD prevention and therapeutic strategies.

## Abstract (web summary)

In Alzheimer’s disease, neurons are considered the sole source of amyloid-β (Aβ) peptides that form plaques. Here the authors show that oligodendrocytes, the myelinating glial cells of the brain, also contribute to Aβ plaque burden alongside neurons.

## Main

In Alzheimer’s disease (AD), amyloid-β (Aβ) production has primarily been attributed to excitatory neurons (ExNs)^1^, despite emerging evidence that other cell types might contribute to Aβ production^2,3^. Cultured oligodendrocytes (OLs) are capable of generating detectable levels of Aβ in vitro^4–6^. Because OL lineage cells are abundantly present in both gray matter and white matter (WM), and myelin alterations have been implicated in AD^7–9^, we asked whether OLs directly contribute to Aβ plaque burden in vivo.

**Fig. 1:**
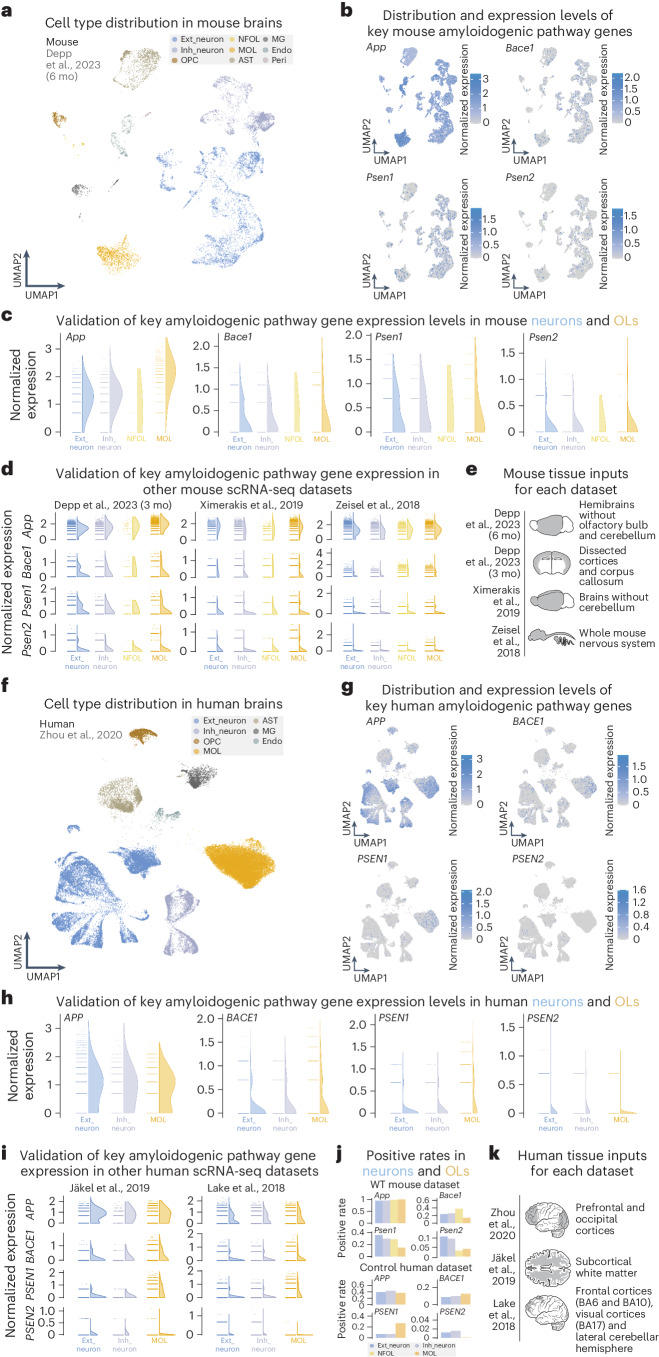
OLs abundantly express key amyloidogenic pathway genes as assessed by scRNA-seq and snRNA-seq. **a**, UMAP visualization of cell types from a 6-month-old mouse brain snRNA-seq dataset^9^. **b**, Feature plots showcasing expression of key amyloidogenic genes (*App*, *Bace1*, *Psen1* and *Psen2*) across all cell types in WT mouse brains. **c**, Expression level half violin plots of key amyloidogenic genes in neurons and OLs of mouse brains normalized by the SCTransform method, highlighting the similar expression of all genes between neurons and OLs. **d**, Expression level half violin plots of key amyloidogenic genes in neurons and OLs normalized by the SCTransform normalization method from additional mouse datasets^9–11^. **e**, Mouse nervous tissue inputs for sequencing from each study are shown. **f**, UMAP visualization of cell types from a human brain snRNA-seq dataset^12^. **g**, Feature plots showcasing expression of key amyloidogenic genes (*APP*, *BACE1*, *PSEN1* and *PSEN2*) across all cell types in control human brains. **h**, Expression level half violin plots of key amyloidogenic genes in neurons and OLs of human brains with the SCTransform normalization method, highlighting the similar expression of these genes between neurons and OLs. **i**, Expression level half violin plots of key amyloidogenic genes in neurons and OLs normalized by the SCTransform normalization method from additional human datasets^13,14^. **j**, Positive rate bar plots of APP processing genes in mouse and human nervous tissue inputs. **k**, Human nervous tissue inputs for sequencing from each study are shown. **c**,**d**,**h**,**i**, Half violins represent aggregated expression levels of respective genes from each cell type, and data points refer to individual expression levels from single cells or nuclei normalized by SCTransform. The results published here are based on data obtained from the Gene Expression Omnibus and the AD Knowledge Portal. mo, months; UMAP, uniform manifold approximation and projection.

**Extended Data Fig. 1:**
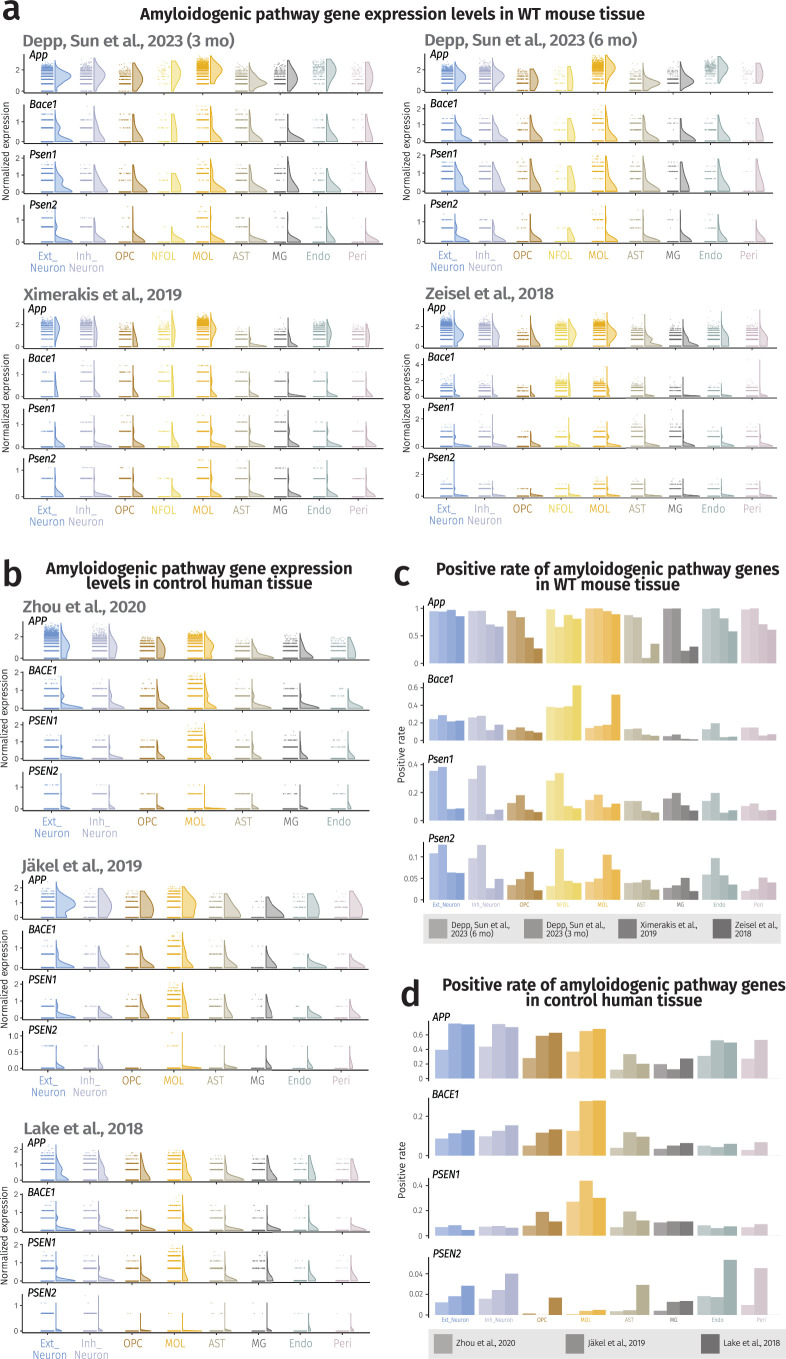
Expression levels and positive rates of amyloidogenic pathway genes across all cell types in the nervous system. (**a**) Expression level half violin plots of amyloidogenic pathway genes in all cell types of mouse nervous tissue inputs with SCTransform normalization method from all chosen mouse datasets^9,26,27^. (**b**) Expression level half violin plots of amyloidogenic pathway genes in all cell types of human nervous tissue inputs with SCTransform normalization from all chosen human datasets^28–30^. (**a, b**) Half violins represent aggregated expression levels of respective genes from each cell type and data points refer to individual expression levels from single cells or nuclei normalized by the SCTransform method. (**c**) Positive rate barplots of amyloidogenic pathway genes in all cell types of mouse nervous tissue inputs from all chosen mouse datasets^9,26,27^. (**d**) Positive rate barplots of amyloidogenic pathway genes in all cell types of human nervous tissue inputs with SCTransform normalization method from all analyzed human datasets^28–30^. The results published here are based on data obtained from GEO and the AD Knowledge Portal. Source data

**Extended Data Fig. 2:**
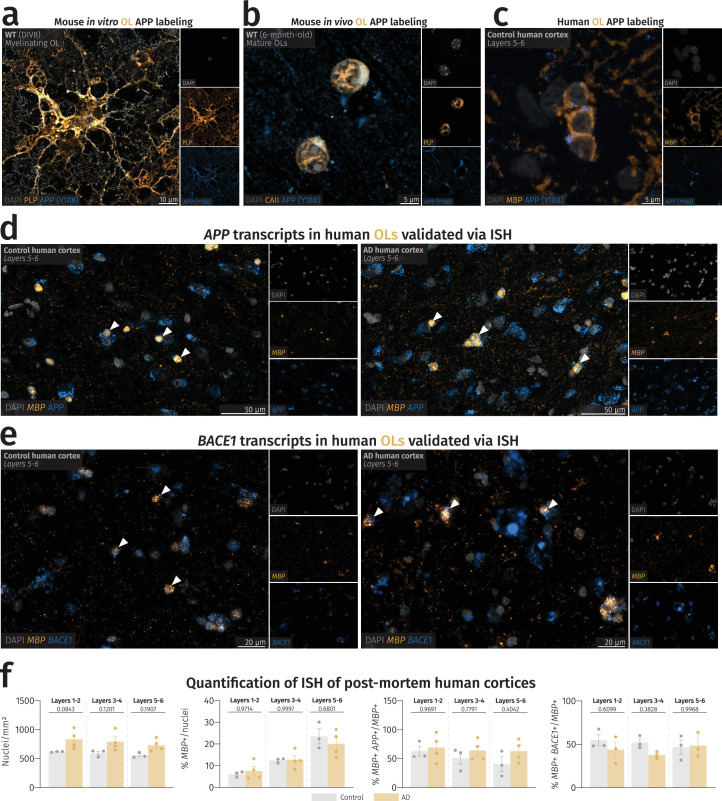
OLs exhibit protein expression of APP and an abundance of *APP* and *BACE1* transcripts. (**a**) A confocal image of an in vitro OL with APP present in the soma and cellular processes. Experiment was repeated thrice in separate in vitro cultures. (**b**) A confocal image of in vivo mouse cortical OLs with APP present in the perinuclear space. Experiment was repeated thrice in separate in vitro cultures. (**c**) A fluorescence microscopy image of human cortical OLs showing APP reactivity in the perinuclear space. Immunolabeling was performed once on all control human samples (n = 3). (**d**) ISH images of human cortical layers 5–6 with visible *APP* puncta in *MBP*+ cells of control (left) and AD (right) patients. Arrowheads point to *APP*-expressing OLs. (**e**) ISH images of human cortical layers 5-6 with visible *BACE1* puncta in *MBP*+ cells of control (left) and AD (right) patients. Arrowheads point to *BACE1*-expressing OLs. (**f**) Quantification of nuclear count, *MBP*+ nuclei, *MBP*+ *APP*+ nuclei, and *MBP*+ *BACE1*+ nuclei in control (n = 3) and AD (n = 3–4) patients. One-way ANOVA was performed with Sidak multiple comparison tests (*P* values indicated in graphs with significance highlighted in bold) comparing AD patients to controls. Bars represent means with SEM, and individual data points are displayed. Source data

We first interrogated multiple sequencing datasets of wild-type (WT) mouse^9–11^ and healthy control human^12–14^ nervous tissue for expression of amyloidogenic pathway genes (*APP*, *BACE1*, *PSEN1* and *PSEN2*) (Fig. 1 and Extended Data Fig. 1a,b). Depending on the sequencing technology and tissue input, positive cell rates of amyloidogenic transcripts varied, but expression levels were similar between neurons and OLs (Extended Data Fig. 1c,d). We validated the expression of amyloid precursor protein (APP) in murine OLs in vitro and in vivo (Extended Data Fig. 2a,b), alongside human OLs via immunolabeling (Extended Data Fig. 2c). By in situ hybridization (ISH) in human cortical tissue, we found that approximately 50% of all gray matter OLs express considerable levels of *APP* and *BACE1* mRNA in both AD cases and controls (Extended Data Fig. 2d–f). Thus, both mouse and human OLs express the essential components for Aβ generation.

**Fig. 2:**
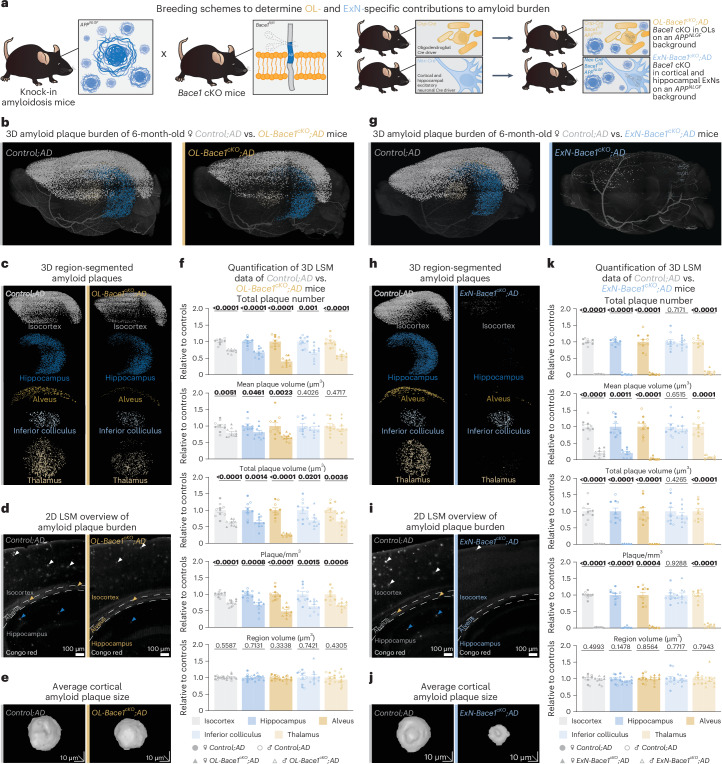
OLs contribute to Aβ burden primarily derived from ExNs in vivo*.* **a**, Mouse breeding setup to investigate the OL-specific and ExN-specific contributions to Aβ burden. **b**–**f**, LSM data of plaque burden (Congo red) comparing 6-month-old *OL-Bace1*^*cKO*^*;AD* mice to age-matched and sex-matched littermate controls. **g**–**k**, LSM data of plaque burden (Congo red) comparing 6-month-old *ExN-Bace1*^*cKO*^*;AD* mice to age-matched and sex-matched littermate controls. **b**–**k**, Color-region allocation is as follows: white, isocortex; blue, hippocampus; yellow, alveus; pastel blue, inferior colliculus; pastel yellow, thalamus. **b**,**g**, LSM 3D visualization of control and cKO hemibrains. **c**,**h**, Brain region-segmented plaques of control and cKO hemibrains. **d**,**i**, LSM 2D single plane of control and cKO hemibrains. Arrowheads point to plaques with colors indicating specific regions. **e**,**j**, LSM 3D renders of representative cortical Aβ plaques of control and cKO hemibrains. **f**,**k**, Quantification of LSM data between controls (*n* = 5 per sex) and cKOs (*n* = 5 per sex). Normalization of cKO data points to sex-matched controls was performed. Circles represent controls, and triangles represent cKOs. Filled shapes represent male mice, and hollowed shapes represent female mice. For each parameter, unpaired, two-tailed Student’s *t*-test was performed (*P* values indicated in graphs with significance highlighted in bold) comparing cKOs to controls. Bars represent means with s.e.m., and individual data points are displayed. Raw data are available in Supplementary Tables 1 and 2. Source data

Next, we created novel AD mouse lines to assess Aβ contribution from OLs and ExNs separately (Fig. 2a). For this, we employed *APP*^*NLGF*^ knock-in mice that express a humanized and triple-mutated *APP* in the endogenous *App* locus to circumvent transgenic mouse artifacts. These mice were crossed with *Bace1*^*fl/fl*^ mice to conditionally knock out *Bace1* (*Bace1* cKO), the rate-limiting enzyme in Aβ generation, using cell-type-specific *Cre* drivers, namely, *Cnp-Cre* for OLs and *Nex-Cre* for dorsal telencephalic ExNs. We termed the resultant triple-mutant mice *OL-Bace1*^*cKO*^*;AD* and *ExN-Bace1*^*cKO*^*;AD*, respectively, and compared them to non-*Cre* controls termed *Control;AD*.

**Extended Data Fig. 3:**
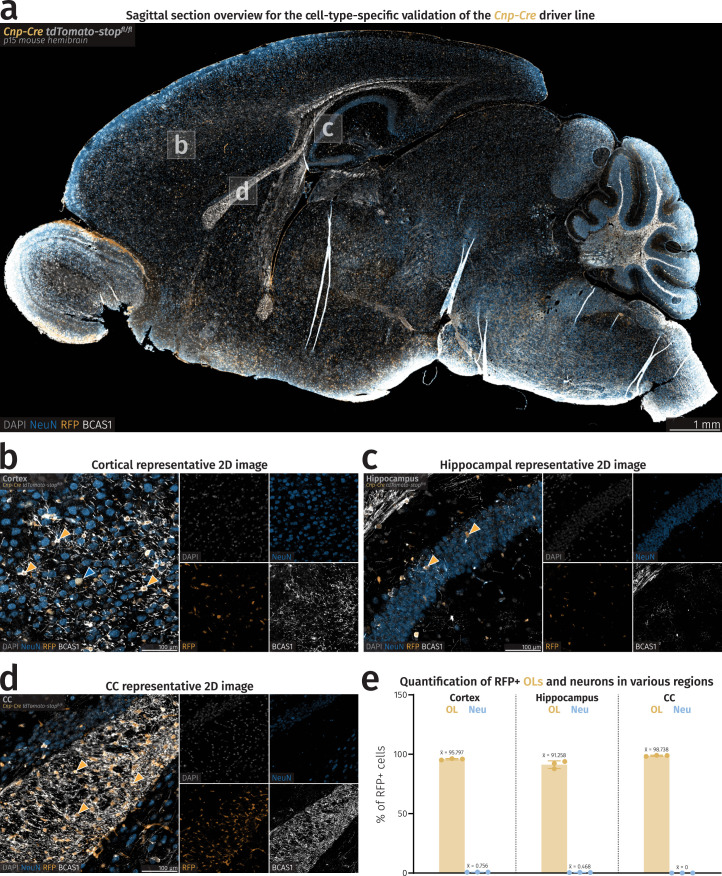
Validation of *Cre* specificity in *Cnp-Cre* stop-flox tdTomato mice. (**a**) Fluorescence microscopy sagittal overview of a *Cnp-Cre* stop-flox tdTomato mouse. (**b-d**) Closeup images of cortex, hippocampus, and CC of a *Cnp-Cre* stop-flox tdTomato mouse. Yellow arrowheads point to RFP+ OLs and the blue arrowhead points to a single RFP+ neuron in the cortex. (**e**) Barplots showing percentages of RFP+ OLs and neurons in specific brain regions. Mean percentage values are shown above each bar. Rounded average total number of cells considered for quantification is as follows: Cortex–OLs=283, cortex–neurons=9,232, hippocampus–OLs=66, hippocampus–neurons=3,203, CC–OLs=344, CC–neurons=1. Source data

**Extended Data Fig. 4:**
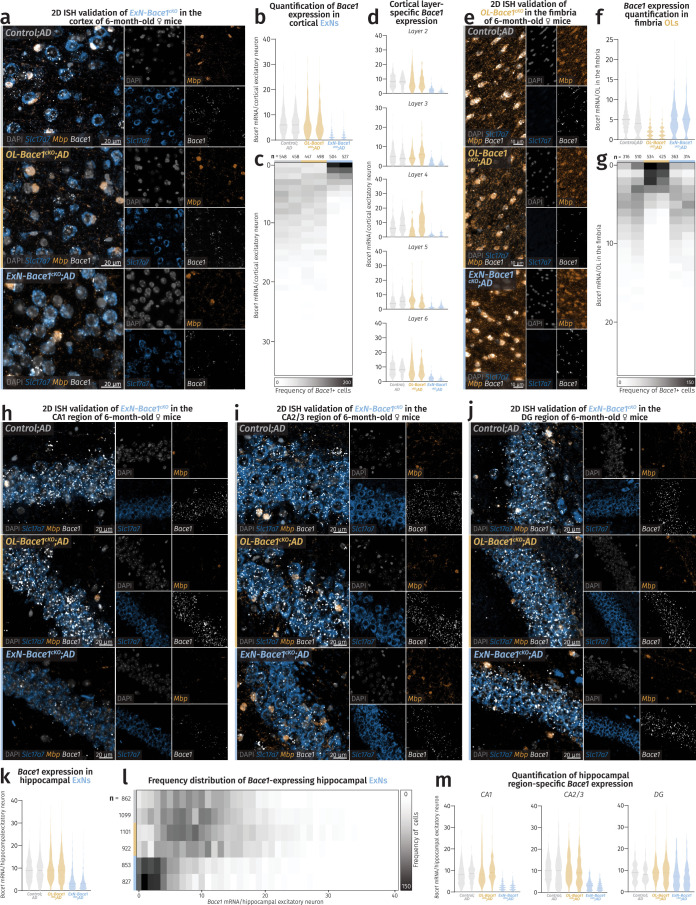
ISH validation of *Bace1* cKO in *OL*-*Bace1*^*cKO*^*;AD* and *ExN*-*Bace1*^*cKO*^*;AD.* (**a-d**) ISH validation of *Bace1* cKO in cortical ExNs. (**e-g**) ISH validation of *Bace1* cKO in fimbria OLs. (**h-m**) ISH validation of *Bace1* cKO in hippocampal ExNs. (**a**) Fluorescence microscopy images of cortices showing reductions of *Bace1* transcripts in ExNs of *ExN-Bace1*^*cKO*^*;AD* samples. (**b**) Violin plots showing individual mouse distribution of *Bace1* puncta/cortical ExN nuclei. (**c**) Frequency distribution heatmap of individual mouse distribution of *Bace1* puncta/cortical ExN nuclei. (**d**) Violin plots showing cortical layer-specific individual mouse distribution of *Bace1* puncta/ExN nuclei. (**e**) Fluorescence microscopy images of hippocampal fimbriae showing reductions of *Bace1* transcripts in OLs of *OL-Bace1*^*cKO*^*;AD* samples. (**f**) Violin plots showing individual mouse distribution of *Bace1* puncta/fimbria OL nuclei. (**g**) Frequency distribution heatmap of individual mouse distribution of *Bace1* puncta/fimbria OL nuclei. (**h-j**) Fluorescence microscopy images of hippocampal CA1, CA2/3, and DG respectively showing reductions of *Bace1* transcripts only in ExNs of *ExN-Bace1*^*cKO*^*;AD* samples. (**k**) Violin plots showing individual mouse distribution of *Bace1* puncta/hippocampal ExN nuclei. (**l**) Frequency distribution heatmap of individual mouse distribution of *Bace1* puncta/hippocampal ExN nuclei. (**m**) Violin plots showing hippocampal region-specific individual mouse distribution of *Bace1* puncta/ExN nuclei. For (**b,d,f,k,m**), comparison was made between control, *OL-Bace1*^*cKO*^*;AD*, and *ExN-Bace1*^*cKO*^*;AD* mice (n = 2 per group). Solid lines represent median and faded lines represent quartiles. For (**c,g,l**), n-numbers refer to amount of nuclei considered for each region and cell type analysis and make up the data cloud for violin plots shown in (**b,d,f,k,m**). Source data

**Extended Data Fig. 5:**
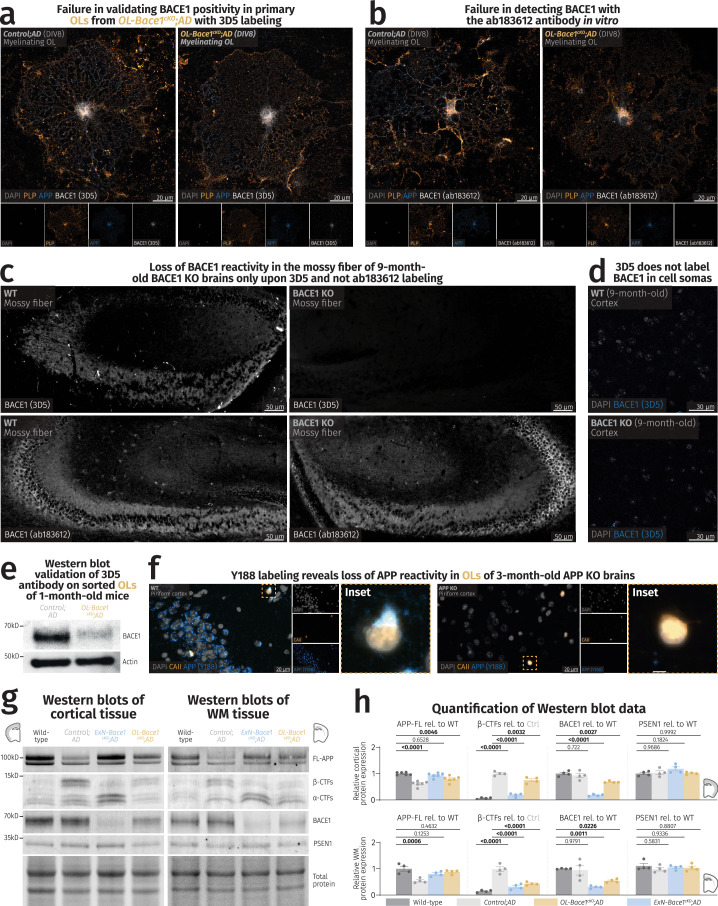
Validation of BACE1 and APP antibodies with KO materials. (**a**) Confocal images of primary OLs harvested from *Control;AD* and *OL-Bace1*^*cKO*^*;AD* mice labeled with PLP (yellow), APP (blue), and 3D5 for BACE1 (white). The punctate 3D5 labeling is equally present in *Control;AD* and *OL-Bace1*^*cKO*^*;AD,* suggesting an unspecific BACE1 staining. Experiment was repeated twice in separate in vitro cultures. (**b**) Confocal images of primary OLs harvested from *Control;AD* and *OL-Bace1*^*cKO*^*;AD* mice labeled with PLP (yellow), APP (blue), and ab183612 for BACE1 (white). No BACE1 reactivity could be detected. Experiment was repeated twice in separate in vitro cultures. (**c**) Fluorescence microscopy images of mossy fibers of 9-month-old WT (left) and constitutive BACE1 KO (right) animals labeled with 3D5 (top) and ab183612 (bottom) for BACE1 (white). Loss of mossy fiber staining was only detected via 3D5 labeling. Immunolabeling was performed once on brain slices from different mice (n = 2 per group). (**d**) Confocal images of 9-month-old WT (top) and constitutive BACE1 KO (bottom) cortices labeled with DAPI (gray) and 3D5 for BACE1 (blue). Note the absence of any intracellular staining of 3D5 in both WT and BACE1 KO animals, hinting at the inability of the 3D5 antibody to detect BACE1 in cell somas. Immunolabeling was performed once on brain slices from different mice (n = 2 per group). (**e**) Immunoblot representative images of BACE1 and the loading control, actin, on sorted OLs from 1-month-old *Control;AD* and *OL-Bace1*^*cKO*^*;AD* (n = 1 per group), showing a proof of concept ablation of BACE1 in a cell-type-specific manner. (**f**) Fluorescence microscopy images of piriform cortices of 3-month-old WT (left) and constitutive APP KO (right) animals labeled with CAII (yellow) and Y188 for APP (blue) for BACE1 (white). APP reactivity, including the APP labeling in OL soma (insets), is abolished in the KO tissue. Immunolabeling was performed once on brain slices from different mice (n = 2 per group). **(g-h) Cell-type-specific deletion of** ***Bace1*** **alters APP processing**. (**g**) Immunoblots and total protein content of microdissected cortical and WM tissues from 6-month-old male mice targeting key amyloidogenic proteins in lysates. (**h**) Immunoblot quantification showing APP processing in WT, control, *ExN-Bace1*^*cKO*^*;AD* and *OL-Bace1*^*cKO*^*;AD* (n = 4–6 per group) lysates. Top–cortical, bottom–WM. All immunoblots were normalized to WT relative protein amount except β-CTFs which were normalized to control *AD* relative protein amount. Data was statistically analyzed via one-way ANOVA was performed with Tukey multiple comparison tests (*P* values indicated in graphs with significance highlighted in bold). Bars represent means with SEM and individual data points displayed. Source data

We assessed *Cnp-Cre* specificity using a stop-flox tdTomato reporter mouse line as transient neuronal *Cnp-Cre* activity has been detected to varying degrees^15,16^. In concordance with recent findings^17^, only a very low percentage of cortical (0.756% ± 0.057%) and hippocampal (0.468% ± 0.111%) neurons were tdTomato^+^ (Extended Data Fig. 3). We then validated the cell-type-specific *Bace1* transcript reduction using ISH, whereby *Bace1* transcripts were massively reduced in the intended target cell type (Extended Data Fig. 4). Notably, we confirmed that ExN *Bace1* transcript levels were unaffected in *OL-Bace1*^*cKO*^*;AD* animals (Extended Data Fig. 4a–d,h–m)*.* We next attempted to demonstrate BACE1 protein expression in OLs and to validate successful knockdown in OLs on a protein level in OL cultures from *Control;AD* and *OL-Bace1*^*cKO*^*;AD* mice (Extended Data Fig. 5a,b). However, we failed to validate *Bace1* cKO via immunocytochemistry using the 3D5 antibody. We, therefore, investigated the specificity and sensitivity of this antibody in tissue sections by comparing WT animals to constitutive *Bace1* KO animals. We only observed a loss of 3D5 labeling in the mossy fibers in *Bace1* KO (Extended Data Fig. 5c), yet no somatic BACE1 was observed (Extended Data Fig. 5d). As immunohistochemical validation of *Bace1* KO was not feasible, we sorted OLs from *Control;AD* and *OL-Bace1*^*cKO*^*;AD* mice and showed abolishment of BACE1 in cKO tissue via immunoblotting by which BACE1 can be readily detected (Extended Data Fig. 5e). We also validated the specificity of the APP antibody Y188 by investigating constitutive APP KO tissue, which revealed loss of both neuronal and OL APP (Extended Data Fig. 5f).

We then turned to western blot analysis to validate APP processing alterations (Extended Data Fig. 5g,h). Full-length APP (FL-APP) levels were 40% lower in control *APP*^*NLGF*^ lysates compared to WT brains. As expected, both *Bace1* cKOs in ExNs and OLs resulted in a region-dependent depletion of BACE1, reflecting local differences in neuron-to-OL ratio. Of note, WM tracts harbor a substantial amount of axoplasm containing neuronally expressed BACE1 (ref. ^18^), explaining the reduction seen in the WM of *ExN-Bace1*^*cKO*^*;AD* mice. Cell-type-specific losses of BACE1 diminished β C-terminal fragments (β-CTFs) in the cKOs and restored FL-APP to nearly baseline WT amounts. Levels of presenilin-1 (PSEN1) remained unchanged.

**Extended Data Fig. 6:**
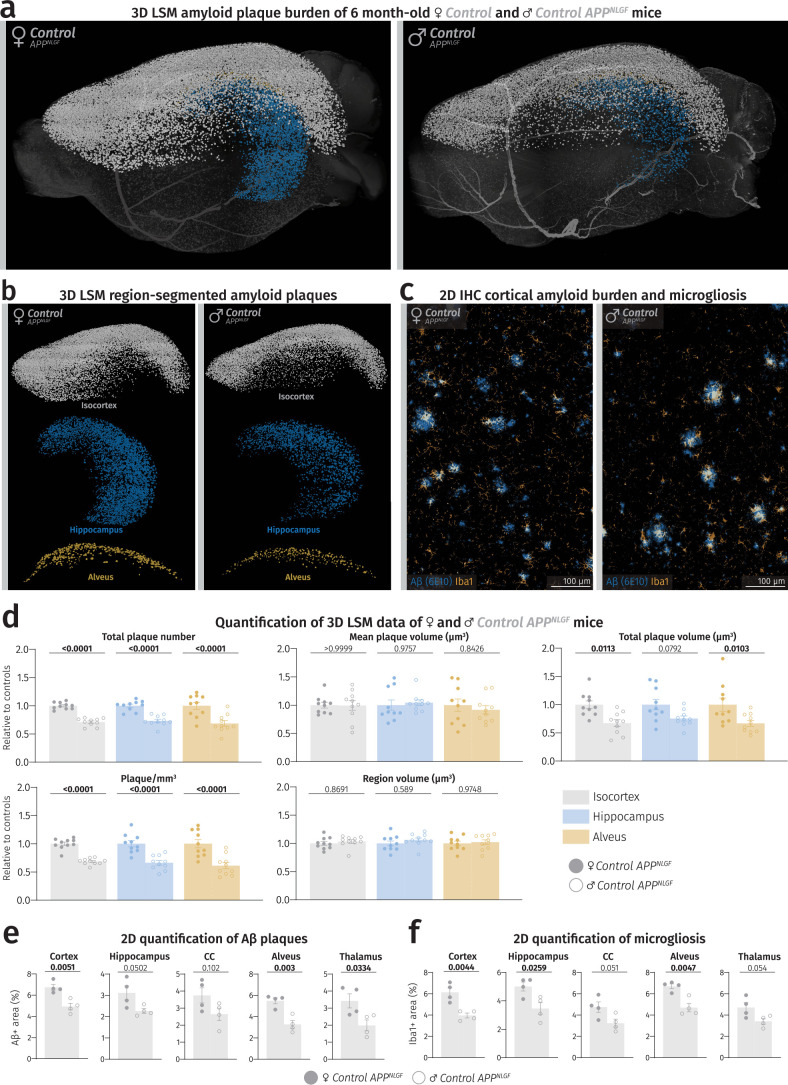
Female *APP*^*NLGF*^ animals develop more Aβ plaque burden compared to age-matched male *APP*^*NLGF*^ animals. (**a**) LSM 3D visualization of female and male control *APP*^*NLGF*^ hemibrains at 6 months of age. (**b**) Brain region-segmented plaques of female and male control *APP*^*NLGF*^ hemibrains. Color-region allocation is as follows: White–isocortex, blue–hippocampus, yellow–alveus. (**c**) Fluorescence microscopy images of female and male control *APP*^*NLGF*^ cortices. (**d**) Quantification of LSM data between female and male control *APP*^*NLGF*^ hemibrains (n = 10 per sex). Male data points were normalized to female data. Filled shapes represent male and hollowed shapes represent female mice. For each parameter, unpaired, two-tailed Student’s *t*-test was performed (*P* values indicated in graphs) comparing males to females. Bars represent means with s.e.m. and individual data points are displayed. Raw unnormalized data is available in Supplementary Table 1. (**e,f**) Quantification of Aβ load and microgliosis in different brain regions of male and female control *APP*^*NLGF*^ mice (n = 4 per sex). Unpaired, two-tailed Student’s *t*-test was performed for each regional quantification (*P* values indicated in graphs with significance highlighted in bold) comparing males to females. Bars represent means with SEM and individual data points displayed. Source data

Next, we used light sheet microscopy (LSM) for in toto imaging of amyloid plaques in *OL-Bace1*^*cKO*^*;AD* and *ExN-Bace1*^*cKO*^*;AD* mouse hemibrains at 6 months in both sexes (Extended Data Fig. 6). We analyzed cortex and hippocampus for gray matter and the alveus as a representative WM tract, alongside the thalamus and inferior colliculus as regions that do not show *Nex-Cre* recombination.

**Extended Data Fig. 7:**
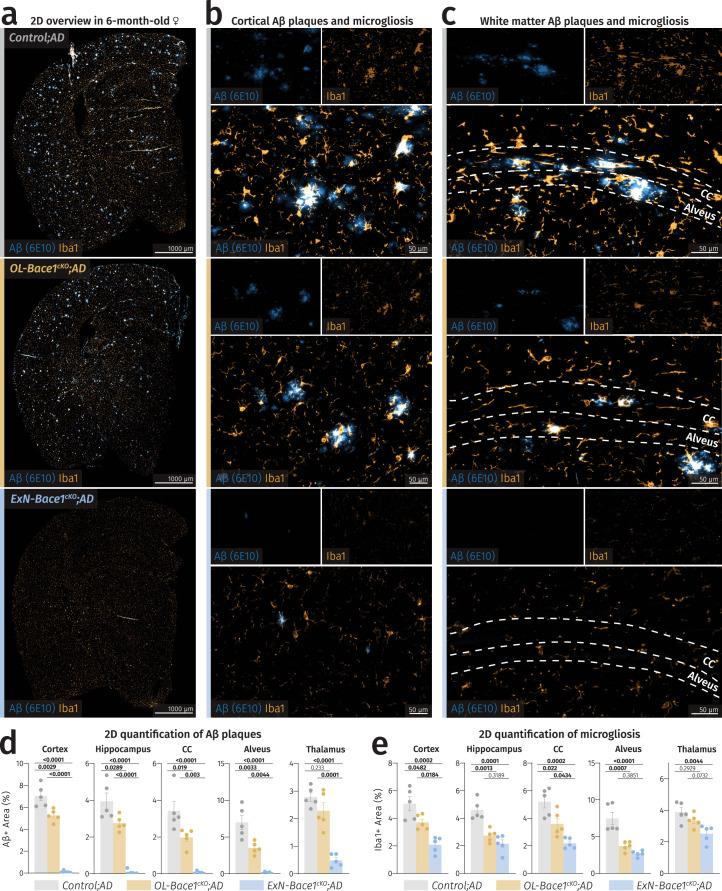
Microgliosis is proportional to Aβ burden. (**a**) Coronal sections of female control, *OL-Bace1*^*cKO*^*;AD* and *ExN-Bace1*^*cKO*^*;AD* mouse hemibrains stained for microglia (Iba1) and Aβ (6E10). (**b,c**) Closeup images of cortex and WM of control and cKO mice showing moderate and marked reductions of both Aβ deposits and microgliosis in *OL-Bace1*^*cKO*^*;AD* and *ExN-Bace1*^*cKO*^*;AD* samples, respectively. Inherent changes in microgliosis could thus be excluded as microglia only appear reactive to plaques and not in regions devoid of them. (**d,e**) Quantification of Aβ load and microgliosis between controls and cKOs (n = 5 per group) spanning different regions. Microgliosis was shown to be directly linked to plaque load. One-way ANOVA was performed with Tukey multiple comparison tests (*P* values indicated in graphs with significance highlighted in bold). Bars represent means with SEM and individual data points displayed. Source data

**Extended Data Fig. 8:**
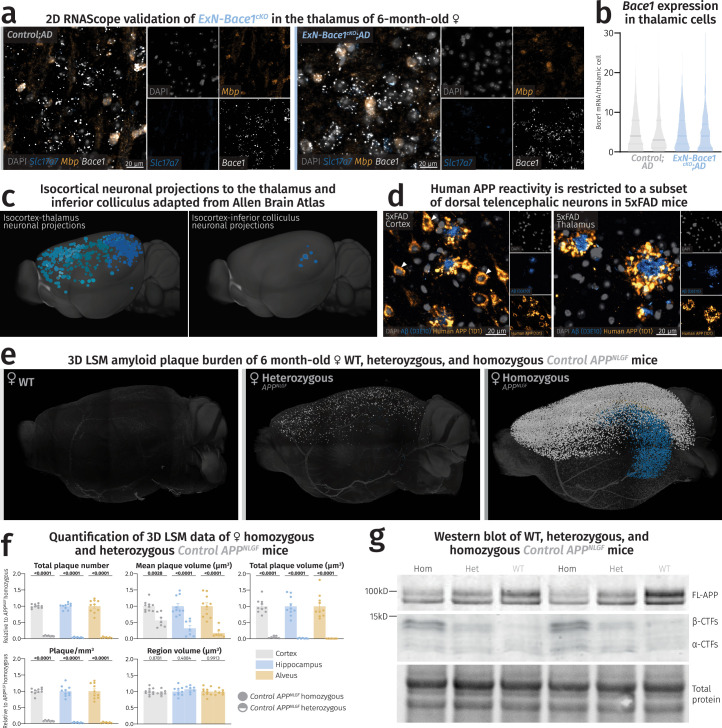
Thalamic plaques contain Aβ derived from cortical and hippocampal sources and plaque deposition is not directly proportional to *APP*^*NLGF*^ gene dosage. (**a**) Fluorescence microscopy images of thalami of control and *ExN-Bace1*^*cKO*^*;AD* samples with no apparent reduction in *Bace1* transcripts in ExNs. (**b**) Violin plots showing individual mouse distribution of *Bace1* puncta/thalamic nuclei. Solid lines represent medians and faded lines represent quartiles. (**c**) Isocortical regions containing neuronal projections into the thalamus (left) and the inferior colliculus (right). Inferior colliculus primarily receives cortical input from the auditory cortex. Images were adapted from the Allen Brain Atlas: Mouse Connectivity: Projection (https://connectivity.brain-map.org/). (**d**) Fluorescence microscopy images of 5xFAD mouse cortex (left) and thalamus (right) stained for Aβ42 (blue) and human-specific APP (yellow). White arrowheads point to neuronal somas in the cortex but not the thalamus of 5xFAD mice positive for human-specific APP. Immunolabeling was performed once on brain slices of 6-month-old 5xFAD mice (n = 4) **(e-g) Plaque deposition is not directly proportional to** ***APP***^***NLGF***^ **gene dosage**. (**e**) LSM 3D visualization of female WT, heterozygous, and homozygous *APP*^*NLGF*^ hemibrains at 6 months of age. (**f**) Quantification of LSM data between female homozygous (n = 10) and heterozygous *APP*^*NLGF*^ mice (n = 7). Heterozygous data points were normalized to homozygous data. Circles represent homozygous *APP*^*NLGF*^ mice and half-filled circles represent heterozygous *APP*^*NLGF*^ mice. For each parameter, unpaired, two-tailed Student’s *t*-test was performed (*P* values indicated in graphs) comparing the two groups. Bars represent means with SEM and individual data points displayed. Raw unnormalized data is available in Supplementary Table 3. (**g**) Immunoblot of 6-month-old male homozygous *APP*^*NLGF*^, heterozygous *APP*^*NLGF*^, and WT mice labeled for APP (n = 2 per group). Heterozygous mice express half of the *APP*^*NLGF*^ gene dosage, resulting in a FL-APP level between WT and homozygous *APP*^*NLGF*^ mice. This is accompanied by a concomitant accumulation of half β-CTFs in heterozygous lysates compared to the homozygous amount. Source data

*OL-Bace1*^*cKO*^*;AD* mice accumulated approximately 30% fewer plaques when compared to respective controls in both sexes (Fig. 2b–f). The decrease in plaque amount and plaque size was greatest in the alveus. Microgliosis was proportional to Aβ plaque pathology (Extended Data Fig. 7). Surprisingly, plaque burden in *ExN-Bace1*^*cKO*^*;AD* mice was reduced by 95–98% compared to controls (Fig. 2g–k), which was much more than anticipated given our findings in *OL-Bace1*^*cKO*^*;AD*. Accordingly, plaque sizes were smaller, and microgliosis was markedly reduced (Extended Data Fig. 7). Moreover, *ExN-Bace1*^*cKO*^*;AD* mice also showed a striking reduction in the amount of thalamic plaques. The unchanged levels of *Bace1* transcript in the thalamus of *ExN-Bace1*^*cKO*^*;AD* mice (Extended Data Fig. 8a,b) indicate that a large amount of subcortical Aβ must be derived from cortico-thalamic axonal projections. Indeed, the inferior colliculus, receiving limited cortical input (Extended Data Fig. 8c) primarily from the auditory cortex^19^, was spared from plaque attenuation in *ExN-Bace1*^*cKO*^*;AD* mice. In fact, immunolabeling of 5×FAD brain slices with the human APP-specific antibody, 1D1, revealed that human APP reactivity in neuronal soma is almost restricted to the cortex (Extended Data Fig. 8d) and hippocampus, confirming the Thy1.2 promoter domain. This further indicates that a subset of dorsal telencephalic neurons is the predominant source of local and distal Aβ plaques.

**Extended Data Fig. 9:**
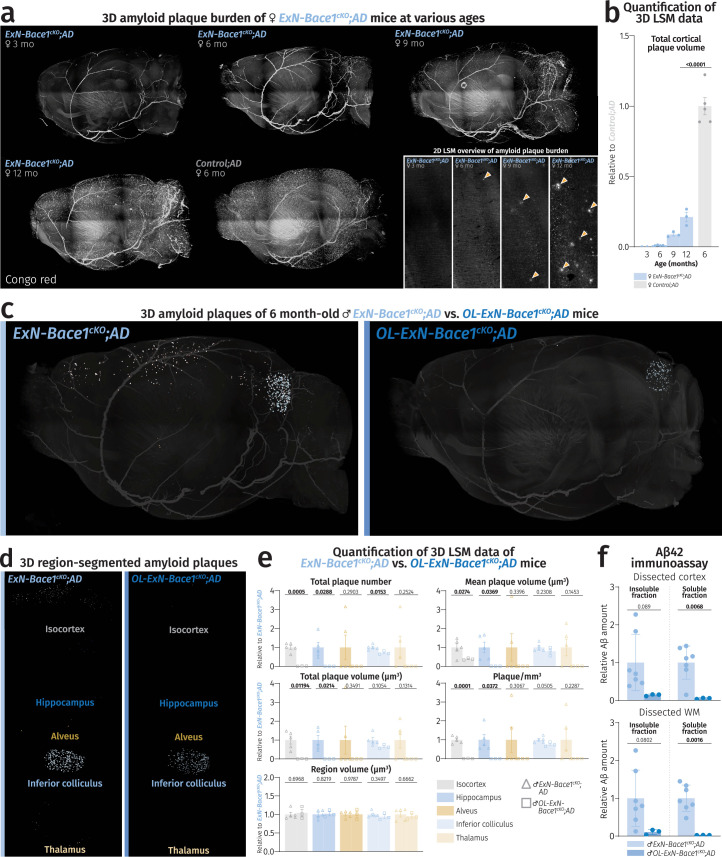
Considerable plaque deposition occurs in older *ExN-Bace1*^*cKO*^*;AD* mice and double *Bace1* cKO in OLs and ExNs ablated cerebral Aβ burden. (**a**) LSM 3D visualization of female *ExN-Bace1*^*cKO*^*;AD* mice at 3, 6, 9, and 12 months alongside a *Control;AD* mouse brain at 6 months stained with Congo Red. Bottom right insets represent 2D cortical planes with yellow arrowheads pointing to plaques. (**b**) Quantification of LSM total cortical plaque volume between *ExN-Bace1*^*cKO*^*;AD* at various time points (n = 3–5 per group). Normalization of *ExN-Bace1*^*cKO*^*;AD* data points to *Control;AD* data points was performed. Blue bars represent *ExN-Bace1*^*cKO*^*;AD* data points and the gray bar represents *Control;AD*. An unpaired, two-tailed Student’s *t*-test was performed (*P* values indicated in the graph with significance highlighted in bold) comparing 12-month-old *ExN-Bace1*^*cKO*^*;AD* and 6-month-old *Control;AD* data. Bars represent means with SEM and individual data points displayed. Raw unnormalized data is available in Supplementary Table 4. **(c-f) Double** ***Bace1*** **cKO in OLs and ExNs ablated cerebral Aβ burden**. Light sheet microscopy data of plaque burden (Congo red) comparing 6-month-old *OL-ExN-Bace1*^*cKO*^*;AD* male mice to age- and sex-matched *ExN-Bace1*^*cKO*^*;AD* mice. Color-region allocation is as follows: White–isocortex, blue–hippocampus, yellow–alveus, pastel blue–inferior colliculus, pastel yellow–thalamus. (**c**) LSM 3D visualization of *ExN-Bace1*^*cKO*^*;AD* and *OL-ExN-Bace1*^*cKO*^*;AD* male mouse hemibrains. (**d**) Brain region-segmented plaques of *ExN-Bace1*^*cKO*^*;AD* and *OL-ExN-Bace1*^*cKO*^*;AD* male mouse hemibrains. (**e**) Quantification of LSM data between *ExN-Bace1*^*cKO*^*;AD* (n = 5) and *OL-ExN-Bace1*^*cKO*^*;AD* (n = 3) hemibrains. Normalization of *OL-ExN-Bace1*^*cKO*^*;AD* data points was performed to *ExN-Bace1*^*cKO*^*;AD*. Hollowed triangles represent *ExN-Bace1*^*cKO*^*;AD* and hollowed squares represent *OL-ExN-Bace1*^*cKO*^*;AD*. For each parameter, unpaired, two-tailed Student’s *t*-test was performed (*P* values indicated in graphs) comparing the two groups. Bars represent means with SEM and individual data points displayed. Raw unnormalized data is available in Supplementary Table 5. (**f**) Aβ42 electrochemiluminescence immunoassay data of insoluble (SDS-soluble, left) and soluble (Tris-NaCl-soluble, right) lysates of microdissected cortical (top) and WM (bottom) tissues from *ExN-Bace1*^*cKO*^*;AD* (n = 7) and *OL-ExN-Bace1*^*cKO*^*;AD* (n = 3) fractions of 6-month-old male mouse hemibrains. Data points were normalized to *ExN-Bace1*^*cKO*^*;AD* samples. The small amount of Aβ42 still produced in *ExN-Bace1*^*cKO*^*;AD* fractions is almost completely ablated in *OL-ExN-Bace1*^*cKO*^*;AD* fractions, highlighting the OL contribution to Aβ42 even in the absence of Aβ42 from ExNs. An unpaired, two-tailed Student’s *t*-test was performed (*P* values indicated in the graph with significance highlighted in bold) comparing the two groups. Bars represent means with SEM and individual data points displayed. Raw unnormalized data are available in Supplementary Table 6. Source data

It was nonetheless puzzling that plaque burden was reduced by more than 95% in *ExN-Bace1*^*cKO*^*;AD* animals, as we had expected that the residual plaque burden would reflect the contribution of OLs (30%). Aβ fibrillation and plaque formation follow sigmoidal growth kinetics^20,21^, and a threshold level of Aβ accumulation is essential for plaque seeding to occur. This threshold level apparently cannot be reached without neuronal Aβ. Fittingly, compared to homozygous *APP*^*NLGF*^ mice, heterozygotes did not develop 50% plaque burden but, rather, less than 10% (Extended Data Fig. 8e–g). This highlights the nonlinear relationship among APP processing, Aβ production and plaque load. Indeed, analysis of 12-month-old *ExN-Bace1*^*cKO*^*;AD* mice revealed considerable plaque deposition (Extended Data Fig. 9a,b), hinting that, with enough time, plaques can still be formed by Aβ from non-ExN sources.

**Fig. 3:**
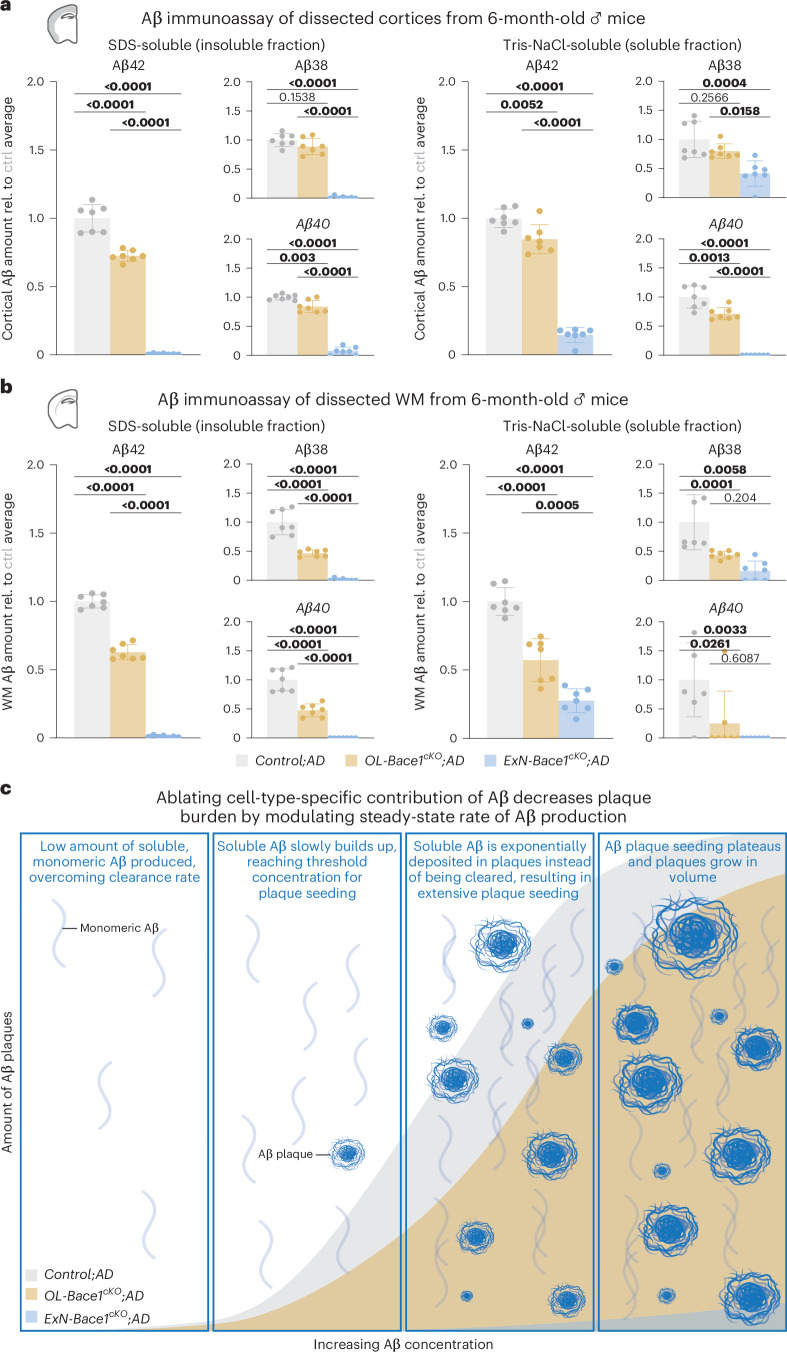
Cell-type-specific deletion of *Bace1* alters the steady-state rate of Aβ production. **a**,**b**, Aβ electrochemiluminescence immunoassay data of insoluble (SDS-soluble, left) and soluble (Tris-NaCl-soluble, right) lysates of microdissected cortical (**a**) and WM (**b**) tissues from control and cKO 6-month-old male mouse hemibrains (*n* = 7 per group). Triplex immunoassay measured Aβ38, Aβ40 and Aβ42 levels, and data points were normalized to *Control;AD* samples. Of note, SDS-soluble fractions from both regions mirrored LSM data, whereas Tris-NaCl-soluble fractions revealed a substantial amount of soluble Aβ still being produced, even in *ExN-Bace1*^*cKO*^*;AD* mice, signifying a residual Aβ production from other cells. Statistical analysis: one-way ANOVA with Tukey multiple comparison tests (*P* values indicated in graphs with significance highlighted in bold). Bars represent means with s.e.m., and individual data points are displayed. Raw unnormalized data are available in Supplementary Table 6. **c**, Working model of modulating cell-type-specific Aβ contributions. Selectively ablating Aβ from specific cell types results in steady-state rate change of Aβ production, causing exponentially slower plaque growth that follows a sigmoidal growth curve. ctrl, control; rel., relative. Source data

Lastly, we performed a sensitive electrochemiluminescence assay for different Aβ species (Aβ38, Aβ40 and Aβ42) to determine total Aβ levels. As inputs, we analyzed soluble and insoluble (representing Aβ primarily bound in plaques) fractions of microdissected cortex for gray matter and corpus callosum (CC) for WM (Fig. 3a,b). *OL-Bace1*^*cKO*^*;AD* brains contained less insoluble and soluble Aβ42 compared to controls, especially in the WM. *ExN-Bace1*^*cKO*^*;AD* brains were almost devoid of insoluble Aβ, but a moderate amount (14.925% ± 0.066%) of soluble Aβ42 was detected in *ExN-Bace1*^*cKO*^*;AD* cortical tissue. Additionally, the residual amount of WM Aβ42 was higher in *ExN-Bace1*^*cKO*^*;AD* brains (27.604% ± 0.072%). In short, although plaque amount was marginally low in the *ExN-Bace1*^*cKO*^*;AD* brains, an adequate amount of soluble Aβ was still generated by non-ExN sources of Aβ, including OLs and potentially other cell types.

To investigate if residual plaques found in *ExN-Bace1*^*cKO*^*;AD* hemibrains are primarily derived from OLs, we generated *Cnp-Cre Nex-Cre Bace1*^*fl/fl*^ *APP*^*NLGF*^ mice, hereby termed *OL-ExN-Bace1*^*cKO*^*;AD*. *OL-ExN-Bace1*^*cKO*^*;AD* developed almost no plaques in the cerebrum (Extended Data Fig. 9c–e). Moreover, *OL-ExN-Bace1*^*cKO*^*;AD* lysates showed an almost complete loss of Aβ42 when compared to *ExN-Bace1*^*cKO*^*;AD* samples (Extended Data Fig. 9f), further highlighting that OLs are a main source of Aβ production even in the absence of the ExN contribution.

**Extended Data Fig. 10:**
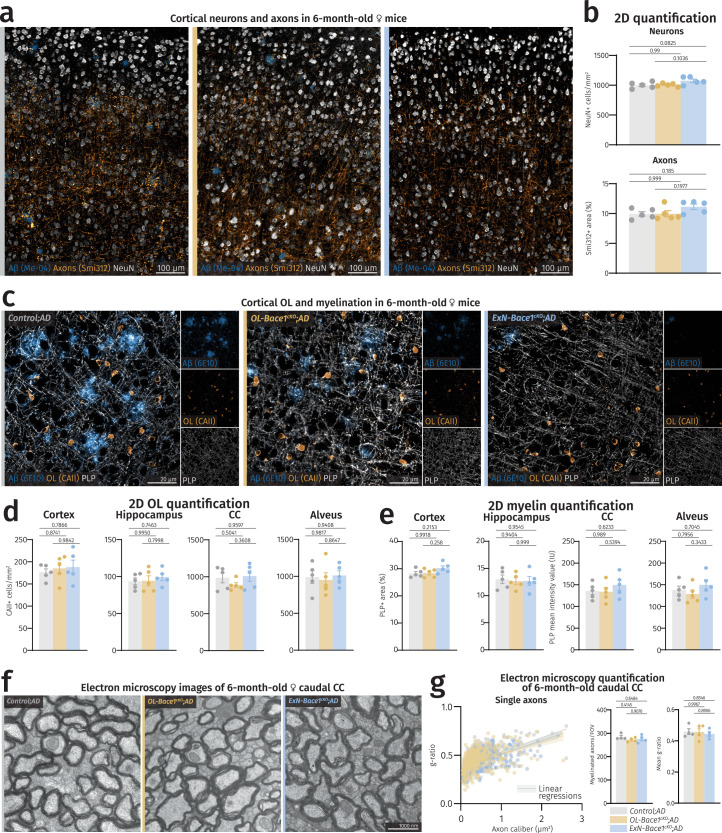
*Bace1* cKO does not result in changes to neuronal numbers, axonal densities, and myelination profile. (**a**) Closeups of fluorescence microscopy images of cortices of controls and cKOs highlighting unchanged density of neurons and axons in both cKO lines at a gross level. (**b**) Quantification of neuronal and axonal densities between 6-month-old female controls and cKOs (n = 5 per group) in the isocortex. **(c-g)** ***Bace1*** **cKO does not alter myelination profile**. (**c**) Closeups of fluorescence microscopy images of cortices of controls and cKOs highlighting unchanged density of OLs and myelination in both cKO lines at a gross level. (**d**) Quantification of OL density between controls and cKOs (n = 5 per group) spanning different regions. (**e**) Quantification of myelin density between controls and cKOs (n = 5 per group) spanning different regions. As CC and alveus are densely myelinated tracts, mean intensity values were instead measured. (**f**) Representative electron micrographs of caudal corpus callosum (CC) of controls and cKOs at 6 months of age. (**g**) Analysis of myelin thickness via g-ratio measurement with single dots representing single myelinated axons quantified (*Control;AD* = 397, *OL-Bace1*^*cKO*^*;AD* = 417, *ExN-Bace1*^*cKO*^*;AD* = 394). Lines represent linear regressions of each group and shaded area indicates error bars. Myelinated axon counts and mean g-ratio comparisons from electron micrographs of controls and cKOs (n = 4 per group). For (**b,d,e,g**), one-way ANOVA was performed with Tukey multiple comparison tests (*P* values indicated in graphs with significance highlighted in bold). Bars represent means with SEM and individual data points displayed. Source data

Functionally, cKO of *Bace1* did not result in any changes to neuronal nor axonal abundance (Extended Data Fig. 10a,b). We also recently showed that OL dysfunction drives neuronal amyloid deposition in AD mouse models^9^. To exclude this as a confounding factor, we compared myelin profiles in *OL-Bace1*^*cKO*^*;AD* and *ExN-Bace1*^*cKO*^*;AD* mice but found no changes (Extended Data Fig. 10c–g).

The high expression level of amyloidogenic pathway genes in OLs was contrasted by the smaller relative contribution to overall Aβ deposition. This could be explained by the differences in number, localization or size between neurons and OLs^22^. There is also the alternative possibility that Aβ processing is more efficient in neuronal compartments or that Aβ isoforms differ between cell types. Beyond their abundance and unique activity profile^23^, however, what makes ExNs so efficient at producing Aβ remains elusive. Identifying the mechanisms that slow down Aβ generation in OLs, despite the abundance of processing substrate and enzymes, could pave the way for novel therapies targeting Aβ generation.

In conclusion, we provide, to our knowledge, the first in vivo evidence that OLs, a glial cell type, are key players in AD—even in the context of establishing primary Aβ pathology. Notably, the 30% plaque reduction in *OL-Bace1*^*cKO*^*;AD* mice falls in the range of effect sizes achieved by aducanumab^24^ and the FDA-approved lecanemab^25^ antibody therapies. Potentially, selective targeting of *BACE1* in OLs could spare the detriments of widespread BACE1 inhibition, especially given the adverse effects seen in BACE1 inhibitor clinical trials^26–28^. Additionally, we showed that ExN-derived Aβ is still required for rapid plaque seeding locally and distally (Fig. 3c). Ultimately, our non-linearity data are relevant when considering anti-Aβ therapeutic interventions, including BACE1 inhibitors, which, as suggested^29,30^, may have potential in preventing amyloidosis before threshold levels are reached.

## Methods

### Reanalysis of single-nucleus RNA sequencing and single-cell RNA sequencing data from mouse and human nervous system

Single-cell/single-nucleus RNA sequencing (snRNA-seq/scRNA-seq) datasets were collected and screened for expressions of *APP*, *BACE1*, *PSEN1* and *PSEN2* across major cell populations in the central nervous system (CNS). In total, four mouse datasets^9–11^ and three human datasets^12–14^ were used. The in-house generated data underwent alignment toward reference genome GRCm38/mm10 using the Cell Ranger toolkit (10x Genomics), where other external datasets were processed from raw count matrices. All data were processed with the R package Seurat (version 4.3.0)^31^ based on original study protocols. Cell type annotations were cross-checked with cluster-specific gene signatures. Afterward, major CNS cell populations, including excitatory neuron (Ext_Neuron), inhibitory neuron (Inh_Neuron), oligodendrocyte precursor cell (OPC), newly formed oligodendrocyte (NFOL), mature oligodendrocyte (MOL), astrocyte (AST), microglia (MG), endothelial cells (Endo) and pericyte, were subset for further screening for APP metabolism-related gene expressions. Each subset dataset underwent renormalization, high variable feature calculation and scaling using the SCTransform pipeline with default parameters. Gene expression levels are visualized in half violin plots using the R package raincloudplots (version 0.0.4)^32^. The positive expression rate of each gene was calculated upon more than one unique molecular identifier (UMI), and the relative proportion is visualized using the R package ggplot2 (version 3.4.4)^33^.

### Mouse models, husbandry and genotyping

All animal experiments were conducted in concordance with German animal welfare practices and local authorities (documentation: 24_KAN_0021_CNCBFL, 24_KAN_0026_NXCBFL and 24_KAN_0024_FFDE). Mice were group-housed in the animal facility of the Max Planck Institute for Multidisciplinary Sciences (MPI-NAT), City Campus, with ad libitum food and regular cage maintenance. All mice were kept under a 12-h dark and 12-h light cycle in an ambient temperature of 21 °C and 45% humidity. All animals are characterized as unburdened, and only organ collection was performed. Mouse strains were kept on a C57BL/6N background. Both sexes were used and indicated in the respective figures. The following mouse strains were used: *APP*^*NLGF*^ (ref. ^34^), *Bace1*^*fl/fl*^ (ref. ^35^), *Cnp*^*Cre/+*^ hereby termed *Cnp-Cre*^36^, *Nex-Cre*^37^, stop-flox tdTomato^38^ and 5×FAD^39^. The crossbreeds generated and analyzed are as follows: *Cnp-Cre Bace1*^*fl/fl*^ *APP*^*NLGF*^ to assess OL-Aβ contribution (*OL-Bace1*^*cKO*^*;AD*); *Nex-Cre Bace1*^*fl/fl*^ *APP*^*NLGF*^ (*ExN-Bace1*^*cKO*^*;AD*) to assess ExN-Aβ contribution; and *Cnp-Cre* stop-flox tdTomato to validate *Cnp-Cre* specificity. *Bace1*^*−/−*^ samples were provided by the laboratory of Robert J. Vassar. *App*^*−/−*^ samples were provided by the laboratory of Ulrike Müller. Ages of animals analyzed are listed on the respective figures. Genotyping was carried out on ear clips from the marking process (see individual strain references for genotyping protocols). Re-genotyping was performed on a small tail biopsy gathered after mice were euthanized. Most experimental cohorts were defined by genotype, and littermate controls were analyzed.

### Mouse tissue extraction

To acquire samples for imaging experiments, animals were euthanized and immediately flushed with cold PBS until the liver was decolorized. Extracted tissues underwent overnight immersive fixation in 4% paraformaldehyde (PFA) in 0.1 M phosphate buffer before switching to PBS for long-term storage at 4 °C. For electron microscopy, perfusion was done with 4% PFA and 2.5% glutaraldehyde in 0.1 M phosphate buffer (K+S buffer) after flushing with PBS and stored at 4 °C long term in 1% PFA in PBS. For biochemical experiments, animals were cervically dislocated, and their brains were quickly extracted before a quick wash in PBS. Tissues were then placed into a custom 1-mm-spaced coronal brain matrix developed in-house (Workshop, MPI-NAT, City Campus) for manual, sequential sectioning with blades. Brain slices were transferred to a glass plate on ice before microdissection of cortices, CC and hippocampi. Dissected tissues were snap frozen and placed in −80 °C until further use.

### Sample preparation and staining for LSM

Fixed tissues were processed for LSM imaging using a modified iDISCO protocol optimized for amyloid plaque staining, as previously reported^9^. Hemibrains were subjected to a sequence of dehydration, autofluorescence quenching, permeabilization and labeling with Congo red dye. After labeling, hemibrains were subjected to a final ascending methanol wash in PBS and overnight incubation in a 1:2 mixture of 100% methanol and dichloromethane (DCM). Finally, the samples were placed in 100% DCM for 1 h 40 min before clearing in ethyl cinnamate (ECI) for imaging.

### In toto LSM imaging and analysis

Cleared hemibrains were imaged with an LSM setup (UltraMicroscope II, LaVision Biotec) with a corrected dipping cap at ×2 objective lens magnification. InspectorPro (version 7.124, LaVision Biotec) software was used to visualize the samples in the mosaic acquisition mode with the following settings: 5-µm light sheet thickness, 20% sheet width, 0.154 sheet numerical aperture, 4-µm z-step size, 2,150 × 2,150 pixels field of view, dynamic focus steps of 5, dual light sheet illumination and 100-ms camera exposure time. Red fluorescence of Congo red-stained hemibrains was recorded with 561-nm laser excitation at 80% laser power and a 585/40-nm emission filter. Image stacks were imported and stitched with Vision4D (version 3.2, Arivis). Regions of interest (ROIs) in this study include isocortex, hippocampus, alveus, inferior colliculus and thalamus. The ROIs were defined based on anatomical landmarks and labeled before segmentation of plaque burden. A machine learning pipeline to extrapolate three-dimensional (3D) shape recognition from two-dimensional (2D) inputs was generated by supplementing 200 desired objects (plaques) and backgrounds (non-plaque structures), respectively. Next, segment co-localization was performed to delineate plaques within specific ROIs. Upon acquiring plaques as voxel objects, noise was removed by deleting objects with voxel sizes 1–10 and plane counts 1–3. Lastly, object metadata and correlated features were exported as spreadsheets.

### Paraffin sample preparation and immunohistochemical staining

Preparation of paraffinized samples and immunohistochemistry (IHC) were performed as previously described^9^. Details of antibodies used for IHC are listed in Supplementary Tables 7 and 8. Nuclei were stained with DAPI (Thermo Fisher Scientific, 300 nM) in PBS. Slides were washed in PBS twice for 5 min and mounted with Aqua-Poly/Mount medium (Polysciences). Finally, slides were left to dry overnight before imaging.

### Vibratome sectioning and immunostaining

Fixed hemibrains were sectioned at 30-µm thickness with a VT 1000S microtome (Leica). Selected vibratome sections were placed into a 12-well plate with 1 ml of TBS in each well. The sections were rinsed three times in PBS for 5 min. For 3D5 immunolabeling, the sections were placed in a 0.1 M sodium citrate pH 9.0 buffer for antigen retrieval. Sections were then incubated in 16 mM glycine in TBS-0.1% Triton solution for 1 h, followed by washing with TBS three times for 5 min each. Blocking was performed in 5% donkey serum in TBS with 0.1% Triton X-100 for 1 h with shaking. Sections were washed twice for 10 min in 1% BSA in TBS with 0.1% Triton X-100 with shaking. Next, the designated primary antibodies (500 µl per well) were applied onto the sections in 1% BSA in TBS-0.1% Triton overnight at 4 °C with shaking. The following primary antibodies were used: anti-BACE1-3D5 (mouse, hybridoma culture supernatant, 1:250) and anti-BACE1-ab183612 (rabbit, Abcam, 1:250). The next day, sections were washed three times for 10 min each in 1%BSA/0.21% Triton-TBS with shaking. Fluorescent secondary antibodies (500 µl per well) were applied in 1% BSA in TBS-0.25% Triton for 2 h in the dark. The following secondary antibodies were used: anti-rabbit Alexa Fluor 555 (donkey/goat, Thermo Fisher Scientific, 1:1,000) and anti-mouse Alexa Fluor 555 (donkey/goat, Thermo Fisher Scientific, 1:1,000). Nuclei were stained with DAPI (Thermo Fisher Scientific, 300 nM) in PBS. Finally, the sections were washed three times for 15 min each in TBS in the dark with shaking. Sections were carefully retrieved from the 12-well plate and mounted with Aqua-Poly/Mount medium (Polysciences). Slides were left to dry overnight before imaging.

### Human tissue collection

Human patient samples (control—one female, two male, age: 74 ± 2.83 years; AD—two female, two male, age: 72.75 ± 1.78 years) were obtained from Neurobiobank Munich with ethical approval from the Ethical Commitee at Ludwig-Maximilians University. Selection of patients was performed upon Braak staging with scores of patients with AD ranging from Braak 5 to 6 and control patient scores ranging from Braak 1 to 3. Postmortem interval of patients ranged between 26 h and 51 h. APOE genotype of all control patients is 3/3, and APOE genotypes of patients with AD are 3/3, 3/4 and 4/4. For ISH experiments, formalin-fixed paraffin-embedded tissue sections were used from human samples.

### ISH

We employed the RNAscope Fluorescent Multiplex assay (ACDBio) for paraffin-embedded samples (5-µm mouse and 4-µm human sections) as previously described^9^. To ensure optimal hybridization, a hydrophobic barrier was created followed by protein digestion via incubation in RNAscope Protease Plus at 40 °C for 15 min (mouse) or RNAscope Protease IV for 20 min (human). The following probes were used: Mm-Mbp (451491-C1), Mm-Bace1-C2 (400721-C2), Mm-Slc17a7-C3 (416631-C3), Hs-MBP-C2 (411051-C2), Hs-APP-C1 (418321-C1) or Hs-BACE1-C3 (422541-C3) at 40 °C for 2 h. For triple visualization of mouse sections, the following fluorophores were applied: Opal 520, 570 and 690, at 40 °C for 30 min. For double visualization of human sections, TSA Vivid Fluorophores (570 and 650) were used. The slides were again washed and stained with DAPI (Thermo Fisher Scientific, 300 nM) for 10 min before mounting with Aqua-Poly/Mount medium (Polysciences).

Upon epifluorescence imaging, validation of *Bace1* deletion in mouse cortical ExNs was performed manually due to the presence of ample satellite OLs in the cortex. A 500-μm-wide ROI spanning all cortical layers was drawn for each coronal brain slice, more specifically in the parietal or somatosensory cortex overlying the hippocampus. For nuclei expressing *Slc17a7*, *Bace1* puncta were quantified to yield individual ExN *Bace1* counts. The data were grouped into distinct cortical layers, which were delineated based on landmarks. Similar manual quantification was also performed on *Mbp*^*+*^ OLs in the fimbria. Validation of *Bace1* deletion in hippocampal ExNs was carried out semi-automatically by creating a pipeline employing a nuclear detection plugin (StarDist) and expanding the captured nuclear ROIs by a pixel size of 10. Combined with particle analyzer and watershed binarization, *Bace1* puncta were detected per hippocampal neuron. For human samples, images of the entire human brain sections were acquired with the PANNORAMIC Midi II Slide Scanner (3DHISTECH) with the ×20 objective and smaller selected regions with the ×40 objective. The human cortex was divided into three areas corresponding to layers 1 and 2 (L1–2), layers 3 and 4 (L3–4) and layers 5 and 6 (L5–6). For each area, 6–12 images of similar size were selected using CaseViewer (version 2.4, 3DHISTECH) and exported via Slide Converter (version 2.3.2, 3DHISTECH). Selected images were randomized using a Fiji filename-randomizer plugin, and counting was done using the Fiji CellCounter plugin.

### In vitro OL culture

OPCs were isolated from p7 mouse brains using magnetic-activated cell sorting (MACS) and anti-NG2 MicroBeads (Miltenyi Biotec). Tissue dissection and cell sorting were performed under sterile conditions. The neural tissue dissociation kit was used according to the manufacturer’s protocol and as described^40^. Dissected brains were transferred into enzyme mix 1, followed by incubation at 37 °C for 15 min. Next, incubation with enzyme mix 2 was done at 37 °C for 20 min with manual dissociation. Tubes were centrifuged at 1,200 r.p.m. for 5 min, and the supernatant was decanted while the pellet was resuspended in DMEM with 1% horse serum. We then passed the cell suspension through a 70-μm and then a 40-μm strainer. The tubes were again centrifuged at 1,200 r.p.m. for 10 min, and the pellet was resuspended and incubated in warm OPC culture medium consisting of 100 ml of NeuroMACS media, 2 ml of MACS NeuroBrew21, 1 ml of penicillin–streptomycin and 1 ml of L-GlutaMAX at 37 °C for 2 h. Next, tubes were centrifuged at 1,200 r.p.m. and 4 °C for 10 min, followed by pellet resuspension and incubation in NG2 MicroBeads diluted in DMEM with 1% horse serum (10 μl of NG2 beads per 10^7^ total cells) at 4 °C for 15 min. The cell suspension was again centrifuged at 1,200 r.p.m. and 4 °C for 10 min, and the supernatant was removed before pellet resuspension in 5 ml of DMEM with 1% horse serum. LS columns (Miltenyi Biotec) were first attached to a magnet before activating with DMEM containing 1% horse serum. The columns were washed three times with DMEM after addition of the cell suspension. The columns were finally detached from the magnet and flushed with 5 ml of DMEM containing 1% horse serum to collect bound cells. Upon detachment, samples were centrifuged at 1,200 r.p.m. for 5 min, and the pellet was resuspended in proliferation medium. OPCs were plated at a density of 1.2 × 10^5^ cells per well on a 12-well plate in proliferation medium, before replacement with OPC differentiation medium at 4 days in vitro (DIV4). Cells were fixed at DIV8 with 4% PFA and washed with PBS three times for 5 min each.

### Immunocytochemical staining

For immunocytochemical labeling, cells were permeabilized with cold 0.3% Triton X-100 in PBS and blocked with 10% goat serum and 0.03% Triton X-100 in PBS for 1 h. Primary antibodies were diluted in 1.5% horse serum in PBS and applied at 4 °C overnight. Coverslips were washed with PBS three times for 5 min and incubated in secondary antibodies diluted in PBS for 1 h. Details of antibodies used for immunocytochemistry are listed in Supplementary Tables 7 and 8. The samples were washed twice for 5 min before incubation with DAPI (Thermo Fisher Scientific, 300 nM) in PBS. Lastly, cells were washed briefly in PBS before mounting with Aqua-Poly/Mount for confocal imaging. All incubation steps were done at room temperature unless stated otherwise.

### Epifluorescence and confocal microscopy

Epifluorescence imaging was carried out with parameters as previously described^9^. Resulting tiled images were stitched in ZEN. For confocal microscopy, images were partially acquired via ZEN software with a Zeiss LSM 800 Airyscan confocal microscope equipped with Plan-Apochromat ×63/1.4 oil DIC M27 objective. Alternatively, images were acquired via LasAF software with a Leica SP8 Lightning confocal microscope equipped with an argon laser and a tuneable white-light laser with ×63/1.4 glycerin objective. Both confocal microscopes are situated at the European Neuroscience Institute and at MPI-NAT, City Campus, respectively.

### Analysis of 2D microscopy images

All 2D image analysis was performed on Fiji (version 1.53c)^41^. For validation of *Cnp-Cre* specificity, thresholding and particle analyzer were performed to segment and quantify neurons, OLs and RFP^+^ cells. Quantification of RFP^+^ OLs in the CC, however, was performed manually due to the dense amount of OLs. Quantification of 2D Aβ and microgliosis was done via thresholding and measurement of positive area. Microscopic analysis of OL numbers between controls and cKOs similarly started with ROI segmentation followed by thresholding and particle analyzer. As for OL numbers in WM tracts, manual quantification was again performed. Finally, percentage ROI area of the cortex and hippocampus occupied by myelinated structures was obtained upon thresholding, and mean intensity values of major WM tracts were measured.

### Electron microscopy

Sample preparation for electron microscopy was performed based on an optimized protocol in the working group^9^. At least ten digital pictures were captured at ×4,000 magnification with a Zeiss EM900 for ultrastructural analysis. Electron micrographs of the caudal CC were analyzed with Fiji. Analysis of g-ratio was conducted as previously described^9^.

### MACS of OLs

OLs were isolated from whole brains (excluding the olfactory bulb and the cerebellum) of 1-month-old mice using an adult brain dissociation kit (Miltenyi Biotec, 130-107-677). OLs were sorted via positive selection by labeling with OL-specific anti-O4 MicroBeads (Miltenyi Biotec, 130-096-670, 1:40). Sorted cells were eluted in 1× PBS containing cOmplete Mini protease inhibitor cocktail (Roche, one tablet per 10 ml of 1× PBS) and PhosSTOP (Roche, one tablet per 10 ml of 1× PBS) and were centrifuged at 13,000 r.p.m. for 5 min. Pellets were snap frozen for further protein analysis and stored at −80 °C. For western blotting, the pellets were resuspended in 28.5 µl of RIPA buffer taken from an aliquot of 10 ml containing one tablet of protease inhibitor and one tablet of phosphatase inhibitor. This was followed by sonication for 3 min in an ultrasonic bath. For loading, 30 µl of 2× Tris-tricine sample buffer (Invitrogen) was added, as was 1.5 µl of 2 M DTT.

### Protein fractionation

Preparation of insoluble and soluble fractions from mouse brain tissue was carried out based on a modified protocol^42^. Tissue homogenization was carried out using a homogenizer (Precellys) on microdissected cortical and CC fractions in reaction tubes containing ceramic beads in cold lysis buffer (pH 8.0) (700 μl for cortex and 500 μl for CC). The following settings were used for the homogenization at 4 °C: 6,500*g* twice for 30 s. The homogenate was carefully transferred to a 1.5-ml reaction tube before spinning with a benchtop centrifuge (Eppendorf) at 17,000*g* and 4 °C for 20 min. The supernatant was collected and served as the soluble protein fraction, while the pellet was resuspended in 2% SDS (500 μl for cortex and 300 μl for CC). The solution was then sonicated on ice for 1 min until the pellet completely dissolved. To remove DNA, 1 μl of benzonase was added into the solution and incubated at room temperature for 5 min. The samples were again centrifuged at 17,000*g* and 4 °C for 20 min before transferring the supernatant to a fresh collection tube, serving as the insoluble fraction. Fractions were stored at −80 °C for further use.

### Western blotting

Only the insoluble fraction was used to probe for APP processing machinery via western blotting. Protein concentration measurement as well as protocol for SDS-PAGE and western blotting with 10–20% gradient gels (Novex) were carried out as previously described^9^. Details of antibodies used for western blotting are listed in Supplementary Tables 7 and 8. For chemiluminescent blots, equal amounts of Western Lightning Plus ECL Oxidizing Reagent Plus and Enhanced Luminol Reagent Plus (PerkinElmer) were first mixed and then applied onto the membrane. To visualize protein with low abundance, SuperSignal West Femto Stable Peroxide and Luminol/Enhancer (Thermo Fisher Scientific) were instead used. Upon washing in TBS-T three times for 10 min each, membranes were fluorescently scanned using an Odyssey platform (LI-COR) or using a ChemoStar imager (Intas) for chemiluminescent visualization. For quantification, background was subtracted, and bands were analyzed using Fiji. Target protein content was normalized to the FastGreen bands of respective controls as indicated in the graphical representations.

### Electrochemiluminescence Aβ immunoassay

To determine Aβ levels in specific brain regions, we turned to the V-PLEX Plus Aβ Peptide Panel 1 (6E10) kit (Meso Scale Discovery (MSD)) and conducted experiments based on instructions provided by the manufacturer. The kit allows multiplex measurement of Aβ38, Aβ40 and Aβ42 from single wells. First, 150 μl of Diluent 35 was added into each well for blocking before the plates were sealed and incubated with shaking at room temperature for 1 h. Each well was subsequently washed three times with 150 μl of wash buffer containing 0.05% Tween 20 in PBS (PBS-T). From a detection antibody solution containing 50× SULFO-TAG anti-Aβ 6E10 antibody diluted in Diluent 100, 25 μl was added into each well, followed by the addition of 25 μl of samples or calibrators per well. The plate was again sealed and incubated with shaking at room temperature for 2 h. Each well was again washed three times with 150 μl of PBS-T (0.05% Tween 20 in PBS) before the addition of 150 μl of 2× Read Buffer T. Lastly, plate measurement was carried out using the MSD QuickPlex SQ 120 reader. In all assays performed, two technical replicates of samples and calibrators were included.

### Data analysis, statistics and figure preparation

All statistical analyses and preliminary graphs were performed with GraphPad Prism 8.0.2. Statistical tests were chosen based on tests for normality. Experimenters were blinded in the analysis of electron microscopy data. Due to the visible effects that *Bace1* cKO has on the plaque load, blinding was not possible for various imaging analyses. No statistical methods were used to pre-determine sample sizes, but sample sizes for primary experiments (that is, quantitative LSM of cKOs and immunoassay) are similar to those shown in our previous publication^9^. The specific statistical analyses performed are listed in the respective figure legends. No animals or data points were excluded from this study. Brain connectivity images were adapted from the Allen Brain Atlas: Mouse Connectivity: Projection (https://connectivity.brain-map.org/)^43^. All figures were prepared with Adobe Illustrator 28.3.

### Reporting summary

Further information on research design is available in the Nature Portfolio Reporting Summary linked to this article.

## Online content

Any methods, additional references, Nature Portfolio reporting summaries, source data, extended data, supplementary information, acknowledgements, peer review information; details of author contributions and competing interests; and statements of data and code availability are available at 10.1038/s41593-024-01730-3.

## Supplementary information

Supplementary Fig. 1. Raw immunoblots shown in this study.

Reporting Summary

Supplementary Tables 1–8 Supplementary Table 1. Raw LSM data of *OL-Bace1*^*cKO*^*;AD* analysis at 6 months (*n* = 5 per sex and genotype) from Fig. 2f. Supplementary Table 2. Raw LSM data of *ExN-Bace1*^*cKO*^*;AD* analysis at 6 months (*n* = 5 per sex and genotype) from Fig. 2k. Supplementary Table 3. Raw LSM data of female heterozygous *APP*^*NLGF*^ analysis at 6 months (*n* = 7) from Fig. 8f. Supplementary Table 4. Raw LSM data of female *ExN-Bace1*^*cKO*^*;AD* analysis at 3 months, 9 months and 12 months (*n* = 3 per age group) from Fig. 9b. Supplementary Table 5. Raw LSM data of male *OL-ExN-Bace1*^*cKO*^*;AD* analysis at 6 months (*n* = 3 per group) from Fig. 9f. Supplementary Table 6. Raw Aβ immunoassay data pertaining to this study. Normalized Aβ immunoassay data of control, *OL-Bace1*^*cKO*^*;AD* and *ExN-Bace1*^*cKO*^*;AD* (*n* = 4 per group) at 6 months of age (Fig. 3). Cortical and WM data are both represented with adjacent columns containing Aβ38, Aβ40 and Aβ42 values. Top half represents measurements of the SDS-soluble fractions, and bottom half represents measurements of the Tris-NaCl-soluble fractions. All values below the detection range of the immunoassay were reported as 0. Supplementary Table 7. Details of primary antibodies used in this study. Supplementary Table 8. Details of secondary antibodies used in this study.

## Source data

Source data Figs. 2 and 3 and Extended Data Figs. 1–10 Source data for Figs. 2 and 3 and Extended Data Figs. 1–10

## Data Availability

The four mouse scRNA-seq/snRNA-seq datasets analyzed were obtained from Depp et al. (GSE178295 and GSE208683)^9^, Ximerakis et al. (GSE129788)^10^ and Zeisel et al. (SRP135960)^11^. The three human scRNA-seq/snRNA-seq datasets were obtained from Zhou et al. (accessed via the AD Knowledge Portal under study snRNAseqAD_TREM2)^12^, Jäkel et al. (GSE118257)^13^ and Lake et al. (GSE97942)^14^. Source data are provided with this paper.

## Code availability

The code used for snRNA-seq is available on GitHub at https://github.com/TSun-tech/2023_Sasmita_OL_BACE.
