# Supplementary material for: Oligodendrocytes produce amyloid-β and contribute to plaque formation alongside neurons in Alzheimer’s disease model mice
Source: Nat Neurosci. 2024 Aug 5;27(9):1668–74. doi: 10.1038/s41593-024-01730-3 (PMC11374705; doi:10.1038/s41593-024-01730-3)

nature neuroscience

Supplementary information https://doi.org/10.1038/s41593-024-01730-3

Oligodendrocytes produce amyloid-β and contribute to plaque formation alongside neurons in Alzheimer's disease model mice

In the format provided by the authors and unedited

Raw immunoblots from Extended Data Fig 5g

Cortex blot 1

Fastgreen (total protein)

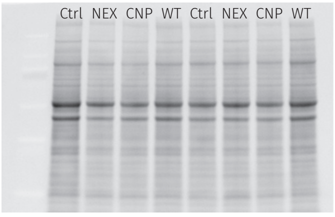

APP-FL (APP, Synaptic Systems)

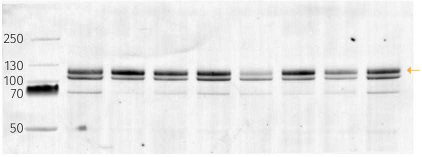

β-CTFs (APP, Synaptic Systems)

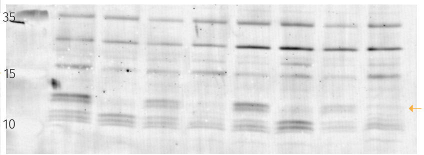

PSEN1 (MAB1563)

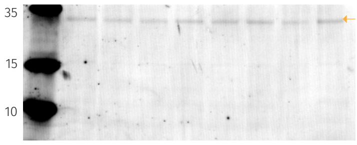

BACE1 (3D5)

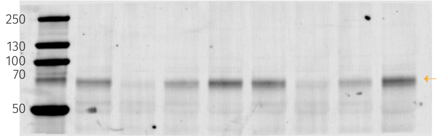

White matter blot 1

Fastgreen (total protein)

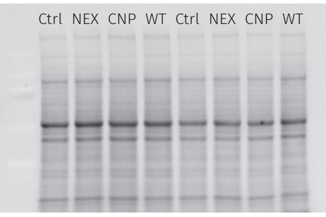

APP-FL (APP, Synaptic Systems)

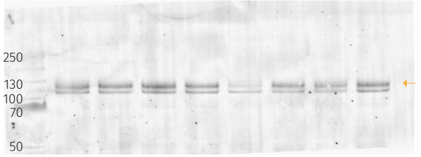

β-CTFs (APP, Synaptic Systems)

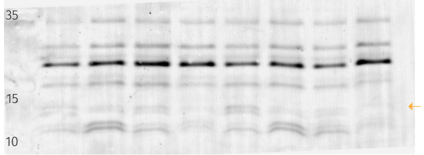

PSEN1 (MAB1563)

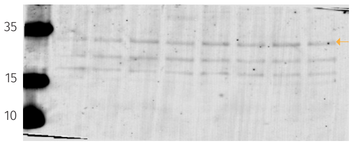

BACE1 (3D5)

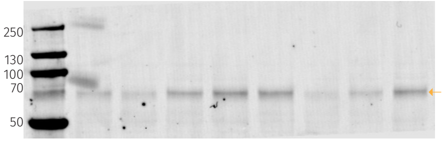

Cortex blot 2

Fastgreen (total protein)

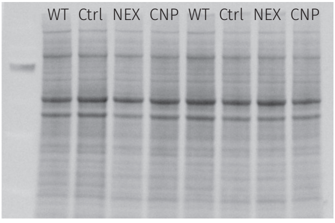

BACE1 (3D5)

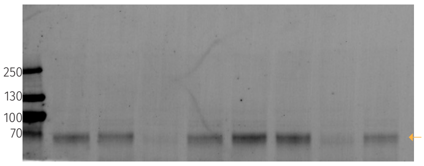

PSEN1 (MAB1563)

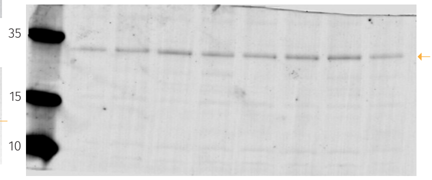

APP-FL (APP, Synaptic Systems)

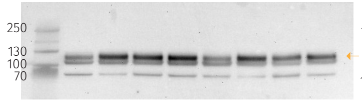

White matter blot 2

Fastgreen (total protein)

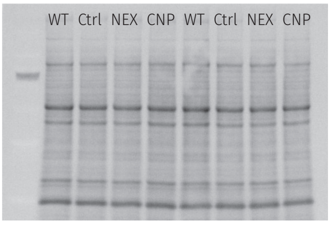

BACE1 (3D5)

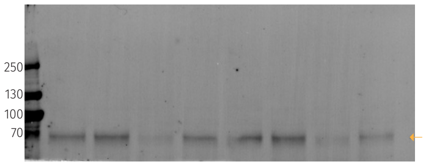

PSEN1 (MAB1563)

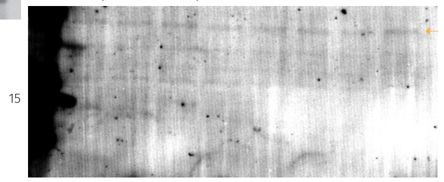

Cortex blot 3

Fastgreen (total protein)

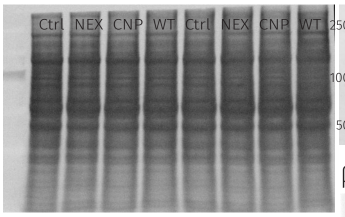

APP-FL (APP, Synaptic Systems)

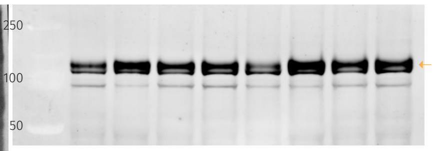

β-CTFs (APP, Synaptic Systems)

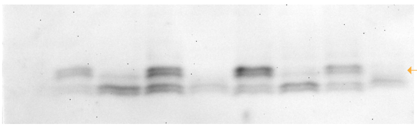

BACE1 (3D5)

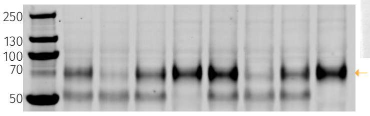

White matter blot 3

Fastgreen (total protein)

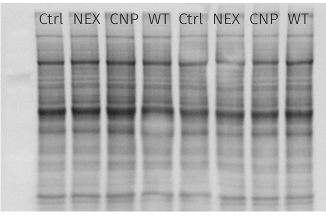

APP-FL (APP, Synaptic Systems)

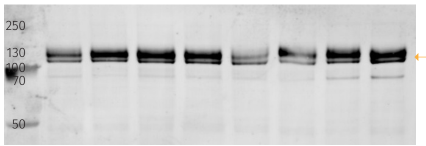

β-CTFs (APP, Synaptic Systems)

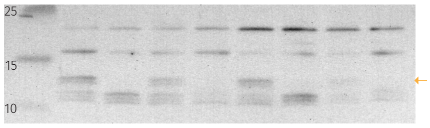

BACE1 (3D5)

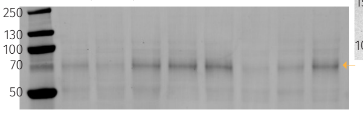

Raw immunoblot from Extended Data Fig 5e

BACE1 (3D5)

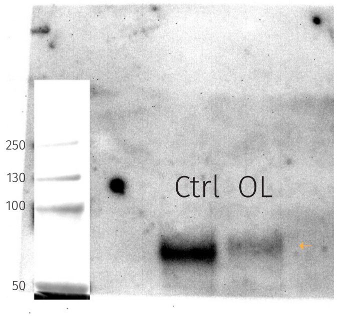

Actin

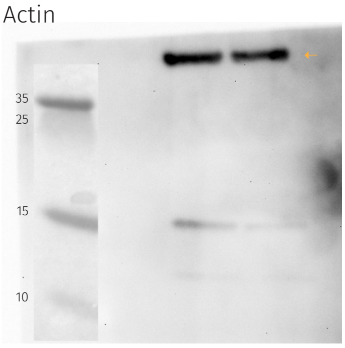

Raw immunoblot from Extended Data Fig 8g

Fastgreen (total protein)

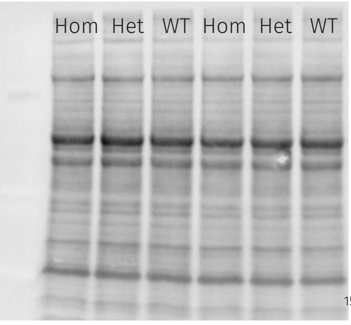

APP-FL (APP, Synaptic Systems)

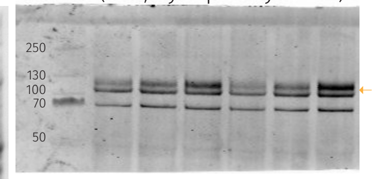

β-CTFs (APP, Synaptic Systems)

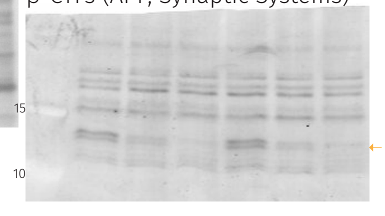

Supplement: Supplementary file 1 — Raw immunoblots shown in this study. [file 41593_2024_1730_MOESM1_ESM.pdf]
