# Supplementary material for: Oligodendrocytes produce amyloid-β and contribute to plaque formation alongside neurons in Alzheimer’s disease model mice
Source: Nat Neurosci. 2024 Aug 5;27(9):1668–74. doi: 10.1038/s41593-024-01730-3 (PMC11374705; doi:10.1038/s41593-024-01730-3)
Supplement: Supplementary file 2 — Reporting Summary [file 41593_2024_1730_MOESM2_ESM.pdf]

nature portfolio

Corresponding author(s): Andrew Octavian Sasmita
Constanze Depp
Klaus-Armin Nave

Last updated by author(s): July 2024

Reporting Summary

Nature Portfolio wishes to improve the reproducibility of the work that we publish. This form provides structure for consistency and transparency in reporting. For further information on Nature Portfolio policies, see our Editorial Policies and the Editorial Policy Checklist.

Statistics

For all statistical analyses, confirm that the following items are present in the figure legend, table legend, main text, or Methods section.

n/a	Confirmed	
☐	☒	The exact sample size (*n*) for each experimental group/condition, given as a discrete number and unit of measurement
☐	☒	A statement on whether measurements were taken from distinct samples or whether the same sample was measured repeatedly
☐	☒	The statistical test(s) used AND whether they are one- or two-sided<br>*Only common tests should be described solely by name; describe more complex techniques in the Methods section.*
☒	☐	A description of all covariates tested
☐	☒	A description of any assumptions or corrections, such as tests of normality and adjustment for multiple comparisons
☐	☒	A full description of the statistical parameters including central tendency (e.g. means) or other basic estimates (e.g. regression coefficient) AND variation (e.g. standard deviation) or associated estimates of uncertainty (e.g. confidence intervals)
☐	☒	For null hypothesis testing, the test statistic (e.g. *F*, *t*, *r*) with confidence intervals, effect sizes, degrees of freedom and *P* value noted<br>*Give P values as exact values whenever suitable.*
☒	☐	For Bayesian analysis, information on the choice of priors and Markov chain Monte Carlo settings
☒	☐	For hierarchical and complex designs, identification of the appropriate level for tests and full reporting of outcomes
☒	☐	Estimates of effect sizes (e.g. Cohen's *d*, Pearson's *r*), indicating how they were calculated

Our web collection on statistics for biologists contains articles on many of the points above.

Software and code

Policy information about availability of computer code

Data collection	Imaging data were acquired using ZEN (v3.3, Zeiss), LAS X (Leica), and ImspectorPro (v. 7_124, LaVision Biotech)
Data analysis	Statistical testing was performed in Prism8.0 (Graphpad) or R (version 4.04, R Core Team 2021). For image analysis: Vision 4D (Arivis, Versions: 3.2, 3.3), FIJI v1.53t (Open source), CaseViewer (3D HISTEC, Versions: 2.3.2, 2.4). For sequencing analysis: CellRanger toolkit v3.0.2 and CellRanger toolkit v6.1.2 (10x Genomics), R package Seurat v3.2.3. and Seurat v4.1.1. scRNA-seq data visualization was assissted by R package raincloudplots (version 0.0.4) and ggplot2 (version 3.4.4). The code used for single-nuclei transcriptome sequencing is available on GitHub https://github.com/TSun-tech/2023_Sasmita_OL_BACE.

For manuscripts utilizing custom algorithms or software that are central to the research but not yet described in published literature, software must be made available to editors and reviewers. We strongly encourage code deposition in a community repository (e.g. GitHub). See the Nature Portfolio guidelines for submitting code & software for further information.

Data

Policy information about availability of data

All manuscripts must include a data availability statement. This statement should provide the following information, where applicable:

- Accession codes, unique identifiers, or web links for publicly available datasets
- A description of any restrictions on data availability
- For clinical datasets or third party data, please ensure that the statement adheres to our policy

All raw sequencing data, as well as raw and processed counts matrices have been uploaded to the Gene expression Omnibus (GEO)101 under the following SuperSeries accession number: The four mouse scRNA-Seq/snRNA-Seq datasets analyzed were obtained from Depp, Sun, et al., 2023 (GSE178295, GSE208683), Ximerakis et al., 2019 (GSE129788), and Zeisel et al., 2018 (SRP135960). The three human scRNA-Seq/snRNA-Seq datasets were obtained from Zhou et al., 2020 (access via AD Knowledge Portal under study snRNAseqAD_TREM2), Jäkel et al., 2019 (GSE118257) , and Lake et al., 2018 (GSE97942). Source data are provided with this paper.

Research involving human participants, their data, or biological material

Policy information about studies with human participants or human data. See also policy information about sex, gender (identity/presentation), and sexual orientation and race, ethnicity and racism.

Reporting on sex and gender	Sexes of patients whose samples are derived from are reported. Sex-specific analysis was not performed due to the small n-number of patient samples. Additionally, experiments involving human samples are proof-of-principle experiments to deduce the expression of amyloidogenic components in human OLs, hence, both sexes are grouped together based on whether they are AD patients or control cases.
Reporting on race, ethnicity, or other socially relevant groupings	N/A
Population characteristics	Human patient samples (Control – 1 female, 2 male, age: 74±2.83 years; AD – 2 female, 2 male, age: 72.75±1.78 years) were utilized for the in situ hybridization experiment. Selection of patients was performed upon Braak staging with AD patient scores ranging from Braak 5-6 and control patient scores ranging from Braak 1-3. Post-mortem interval of patients ranged between 26-51 h. APOE genotype of all control patients are 3/3, while AD patient APOE genotypes are: 3/3, 3/4, and 4/4.
Recruitment	All samples were obtained from the Neurobiobank Munich (Germany).
Ethics oversight	Ethical approval for the use of human post-mortem material was received from the Ethical Commitee at the Ludwig-Maximilians University in Munich, Germany.<br>The brainbank itself has ethical approval to collect post-mortem material to be used for scientific purposes. From all donors or their next of kin informed consent has been obtained.

Note that full information on the approval of the study protocol must also be provided in the manuscript.

Field-specific reporting

Please select the one below that is the best fit for your research. If you are not sure, read the appropriate sections before making your selection.

☒ Life sciences ☐ Behavioural & social sciences ☐ Ecological, evolutionary & environmental sciences

For a reference copy of the document with all sections, see nature.com/documents/nr-reporting-summary-flat.pdf

Life sciences study design

All studies must disclose on these points even when the disclosure is negative.

Sample size	Precalculations of sufficient sample size was not possible as biological effect sizes of the various experimental interferences could not be predetermined. Sample size dependent on availability of mice and previous experiences in regards to histological assessments, analysis of sequencing data etc. No statistical methods were used to pre-determine sample sizes but sample size for primary experiments (i.e., quantitative light-sheet microscopy of cKOs and immunoassay) are comparable to those shown in our past publication (Depp, Sun, et al., 2023).
Data exclusions	No animal or data points were excluded from this study.
Replication	Individual mice were seen as replicates in the case of microscopic analysis, Western blotting, immunoassay, and sequencing analysis. Representative images for qualitative inferences were repeated from at least 2 separate in vitro cultures or 2 separate animals per group to successfully validate antibody specificity and knockouts.
Randomization	In our study, most experimental cohorts were defined by genotype and littermate controls were analyzed.

Blinding	Experimenters were blinded to genotype while performing image analysis. In some cases, genotype of the analyzed animals can be inferred due to gross morphological changes in amyloid plaque content, with conditional knock-outs having less plaque burden.

Reporting for specific materials, systems and methods

We require information from authors about some types of materials, experimental systems and methods used in many studies. Here, indicate whether each material, system or method listed is relevant to your study. If you are not sure if a list item applies to your research, read the appropriate section before selecting a response.

Materials & experimental systems

n/a	Involved in the study
☐	☒ Antibodies
☒	☐ Eukaryotic cell lines
☒	☐ Palaeontology and archaeology
☐	☒ Animals and other organisms
☒	☐ Clinical data
☒	☐ Dual use research of concern
☒	☐ Plants

Methods

n/a	Involved in the study
☒	☐ ChIP-seq
☒	☐ Flow cytometry
☒	☐ MRI-based neuroimaging

Antibodies

Antibodies used	Primary antibodies used (Anti-): 6E10 (mouse, 1:1000, BioLegend, 803001), Actin (mouse, 1:1000, Sigma, A3853), APP-1D1 (rat, 1:500, Sigma, MABN2287), APP-Y188 (rabbit, 1:500, Abcam, ab32136), BACE1 (rabbit, 1:250, Abcam, ab183612), BACE1-3D5 (mouse, 1:250, Gifted by Vassar lab, culture supernatant), BCAS1 (guinea pig, 1:250, Synaptic Systems, 445 003), CAII (rabbit, 1:1000, Abcam, ab124687), Iba1 (rabbit, 1:500, Wako, 019-19741), NeuN (chicken, 1:500, Aves, NUN), PLP-aa3 (rat, 1:200, Generated in-house, culture supernatant), RFP (rabbit, 1:500, Rockland, 600-401-379), APP (rabbit, 1:500, Synaptic Systems, 127 003), PSEN1 (rat, 1:500, Sigma, MAB1563).<br><br>Secondary antibodies used (Anti-): guinea pig DL 650 (Goat, 1:1000, Invitrogen, SA5-10097), mouse Alexa 555 (Donkey, 1:1000, Invitrogen, A-31570), mouse DL 488 (Goat, 1:1000, Dianova, 115-485-003), mouse HRP (Goat, 1:10000, Dianova, 115-035-003), mouse IRDye 680 (Goat, 1:5000, Licor, 926-68070), rabbit Alexa 488 (Donkey, 1:1000, Invitrogen, A-21206), rabbit Alexa 555 (Donkey, 1:1000, Invitrogen, A-31572), rabbit DL 650 (Donkey, 1:1000, Invitrogen, SA5-10041), rabbit HRP (Goat, 1:10000, Dianova, 111-035-003), rabbit IRDye 800 (Goat, 1:5000, Licor, 926-32211), rat DL 488 (Goat, 1:1000, Invitrogen, SA5-10018), rat DL 650 (Donkey, 1:5000, Invitrogen, SA5-10029).<br><br>Full details on antibodies utilized in this study and their experimental usage can be found in Table S7 and Table S8.
Validation	Wherever applicable, validation of antibodies on APPNLGF or corresponding conditional Bace1 knock-outs was performed. Staining or immunoblotting performance was evaluated based on comparison to typical staining patterns that should be observed in APPNLGF animals according to published studies (e.g. staining of amyloid plaques or corralling of glial cells, upregulation in APPNLGF mice). These antibodies were utilized for validation: anti-Aβ-6E10 (mouse, BioLegend, 1:1000); anti-Iba1 (rabbit, Wako, 1:500).<br><br>Validation of Bace1 conditional knock-outs were perfromed via in situ hybridization, Western blotting with the antibody anti-BACE1-3D5 (mouse, hybridoma culture supernatant, 1:500), and immunoassay of amyloid beta on the Meso Scale Discovery platform.<br><br>Myelin protein specific antibody, anti-PLP-clone aa3 (rat, culture supernatant; 1:200), was validated in house against the corresponding knock-out animal (data not shown).

Animals and other research organisms

Policy information about studies involving animals; ARRIVE guidelines recommended for reporting animal research, and Sex and Gender in Research

Laboratory animals	This study involved mice on the C57/BL6N background. Transgenic/genetically modified animals used were: APPNLGF (Saito et al., 2014), Cnp-Cre (Lappe-Siefke et al., 2004), Nex-Cre (Goebbels et al., 2006), stopflox-tdTomato (Madisen et al., 2012), Bace1fl/fl (Hu et al., 2018), 5xFAD (Oakley et al., 2006). This study employed mice of various ages (i.e. for primary cell culture and imaging experiments) as indicated in the figures throughout the paper (p7 to 6-month-old) (Documentation: 24_KAN_0021_CNCBFL, 24_KAN_0026_NXCBFL, 24_KAN_0024_FFDE). Mice were group-housed in the animal facility of Max Planck Institute for Multidisciplinary Sciences (MPI-NAT), City Campus with ad libitum food and regular cage maintenance. All mice were kept under a 12 h dark and 12 h light cycle in an ambient temperature of 21C and 45% humidity. All animals are characterized as unburdened and only organ collection was performed.
Wild animals	This study did not involve wild animals.
Reporting on sex	Both sexes were utilized in this study as sex dimorphism in the APPNLGF mice is evident. When sex dimorphism is expected, such as

Reporting on sex	the light-sheet characterization of plaque burden conditional knockouts, experimental readout was assessed for both sexes. For other experiments, to reduce number of animals utilized, only one sex is chosen as changes persist regardless of sex. Sexes of animals utilized are listed in the figure and respective figure legends.
Field-collected samples	This study did not include field-collected samples.
Ethics oversight	All animal experiments were conducted in concordance with German animal welfare practices and local authorities (Documentation: 24_KAN_0021_CNCBFL, 24_KAN_0026_NXCBFL, 24_KAN_0024_FFDE).

Note that full information on the approval of the study protocol must also be provided in the manuscript.

Plants

Seed stocks	N/A
Novel plant genotypes	N/A
Authentication	N/A
